# How GAI Shapes Travelers’ Booking Decisions: The Influence of Source Disclosure and Valence of Creative Reviews

**DOI:** 10.3390/bs16081417

**Published:** 2026-08-18

**Authors:** Rui Chen, Hao Huang, Xiaoying Qiao

**Affiliations:** 1 Research Institute of International of Economics and Management, Xihua University, Chengdu 610039, China; chenrui@xhu.edu.cn; 2 School of Literature and Journalism, Xihua University, Chengdu 610039, China; cnhuanghao@stu.xhu.edu.cn

**Keywords:** generative artificial intelligence, temporal disclosure, online hotel booking, information adoption model

## Abstract

AI-generated reviews have become a core tool for hotel booking platforms in assisting user decision-making, yet existing research has not systematically explored the emotional tendencies of GAI reviews or the potential mechanisms by which temporal disclosure influences user decisions. This study integrates the Information Adoption Model (IAM) and the Elaboration Likelihood Model (ELM), incorporates the affordance perspective of GAI, employs a 2 × 2 experimental design (temporal disclosure × review valence), analyzes sample data from 649 travelers, and conducts empirical analysis using Partial Least Squares Structural Equation Modeling (PLS-SEM). Results demonstrate that temporal disclosure significantly enhances cognitive involvement; the effect of review valence on cognitive involvement is moderated by GAI affordance—high GAI affordance mitigates the inhibitory impact of positive reviews on cognitive involvement; empathy toward GAI information does not directly reduce psychological distance to hotels but operates indirectly through full mediation via psychological distance to GAI; psychological distance to GAI also indirectly influences booking intent via full mediation through psychological distance to the hotel. This study elucidates the mechanisms linking the emotional characteristics of AI-generated reviews with the impact of temporal disclosure on traveler decision-making, supplements theoretical frameworks for consumer decision-making in AI contexts, expands the IAM for AI applications, and provides empirical evidence and practical recommendations for optimizing GAI review presentation strategies on booking platforms.

## 1. Introduction

Against the backdrop of generative artificial intelligence (GAI) deeply penetrating the hotel and tourism industry, AI-generated hotel reviews, stay guides, and recommendations have become a core source of information for consumers’ booking decisions. Existing research has confirmed that the quality of arguments and source characteristics in online reviews influence users’ information adoption and consumption decisions. However, when it comes to GAI, a new type of information producer, its technological affordances are a significant influencing factor. In the context of AI-generated hotel reviews, a comprehensive theoretical framework explaining how technical features interact with information content to influence users’ information adoption and decision-making has yet to be established. This study adopts the Information Adoption Model (IAM) as its overarching framework, incorporates the dual-path processing logic of the Elaboration Likelihood Model (ELM), and integrates the theory of GAI technological affordances with the theory of psychological distance to construct a model of consumer information adoption and decision-making regarding AI-generated hotel reviews. The study addresses a core question: How does GAI-generated hotel information, through the dual stimuli of content quality and technological affordance, trigger users’ psychological distance from both the hotel and the GAI via cognitive and affective information processing pathways, thereby driving information adoption and converting it into hotel booking intent?

Customer reviews significantly influence hotel booking decisions. TripAdvisor (2023) reports that 93% of travelers rely on reviews to choose the right accommodation. However, too many customer reviews can create information overload, which hinders travelers’ ability to make booking decisions efficiently (S. J. Jia et al., 2025a). To address this challenge, platforms such as TripAdvisor and Airbnb have begun using generative artificial intelligence (GAI) to summarize and distill large volumes of customer reviews, present key information to users, and improve travelers’ decision-making efficiency (Amazon, 2023; Mehta, 2023).

Existing frameworks for IAM research are overly simplistic and fail to account for information processing (Elwalda et al., 2022). While previous studies have focused on user reviews, some researchers have begun to explain users’ willingness to interact with GAI by integrating models such as the Technology Acceptance Model or the Elaboration Likelihood Model (Bahcekapili & Boztas, 2025; Mladenovic et al., 2024). However, in the context of AI-generated hotel reviews—where AI can refine human-generated reviews—using this emerging technology requires examining how perceived differences in its technical characteristics influence user behavior. Research on the adoption of AI-generated content typically considers only informational characteristics, neglecting the significant influence of GAI affordances and the detailed information adoption decision-making process (Yu & Li, 2022). Unlike previous studies that mechanically applied the Information Adoption Model (IAM) to online reviews, this study refines the IAM framework within the context of generative AI. Specifically, in examining how AI-generated hotel reviews influence users’ information adoption and decision-making mechanisms, this study builds upon the IAM framework by breaking down the “argument quality” dimension into two sub-dimensions—temporal disclosure and review valence—and treating the “source credibility” dimension as an affordance of GAI technology, further subdividing it into perceived transparency and perceived creativity. In exploring the information adoption process, this study identified dual pathways of cognitive and affective processing through the relationships among variables, consistent with the theoretical framework of the Elaboration Likelihood Model (ELM). From a theoretical integration perspective, in the context of AI-generated hotel reviews, the adoption of AI review information is not a single process but involves dual cognitive and affective pathways. Therefore, this study introduces the ELM to explain the information processing mechanisms within the IAM, detailing the information processing pathway: argument quality and GAI affordance influence psychological distance through cognitive and affective processing (cognitive involvement and affective involvement—cognitive empathy and affective empathy), which ultimately affects booking intention.

Academic conclusions regarding dual cognitive and affective processing pathways in human–AI interaction remain inconsistent. While existing research has found that both cognitive and affective trust play significant roles in the acceptance of AI services (H. Kim et al., 2025), other empirical studies have shown that the cognitive pathway is more powerful than the affective pathway in motivating users to utilize technology to find innovative solutions (X. Lu & Kim, 2025). This inconsistency, coupled with the complexity of human–AI interaction as an emerging research field, calls for further empirical validation of the dual cognitive and affective processing pathways (Bai et al., 2025).

While existing research has established empathy’s explanatory role in information processing, a comprehensive pathway linking it to behavioral decision-making remains lacking (Gupta & Mukherjee, 2026). Empathy is regarded as a core psychological mechanism in information processing and decision-making research and can serve as a mediating factor in interactive corporate social responsibility (CSR) communication, linking informational stimuli to social identification (Sung & Lee, 2026). In most studies, scholars typically treat empathy as an outcome variable of information processing or a direct predictor of behavioral attitudes (M. Liu & Wu, 2025; Seo et al., 2026). Kushwaha and Kamila (2026) found that the lack of empathy in chatbots led to a loss of perceived value, confirming the psychological role of empathy in decision-making; however, the intermediate mechanisms through which empathy transcends cognitive evaluation to translate into concrete adoption decisions have not yet been systematically elucidated. Although the studies by G. Yang et al. (2026) and Y. Zhao et al. (2026) address both cognitive and affective pathways, they primarily frame empathy within the context of affective processing. In the context of GAI hotel review processing, it is necessary to explore the explanatory power of empathy mechanisms in the psychological transformation process from information processing to adoption decisions.

Therefore, this study aims to explore the primary mechanisms through which the valence of GAI reviews and the timing of their disclosure influence consumer information adoption in online hotel booking scenarios, and proposes the following research questions: (1) Do differences in users’ perceptions of GAI’s technical capabilities (GAI affordance) influence information adoption and, if so, how? (2) Do users experience both cognitive and affective processing pathways simultaneously when processing GAI reviews? (3) Can the empathy mechanism explain the psychological transformation process from information processing to adoption decisions?

To rigorously examine the complex psychological mechanisms underlying travelers’ decision-making in scenarios where GAI generates hotel reviews, this study adopted a methodological framework that combines experimental design with structural equation modeling, rather than relying solely on a single experimental method. Although experimental designs are highly effective in establishing causal relationships between stimuli and immediate responses, a single experimental method often fails to fully elucidate the complex chain of internal psychological processes (Iacobucci et al., 2007). By incorporating PLS-SEM, this study was able to estimate multiple mediating paths simultaneously, thereby revealing the information processing process in greater detail (X. Zhao et al., 2010). Specifically, this mixed-methods approach, combining experimental design with structural equation modeling, enables the testing of continuous mediating paths (Y. Xu et al., 2024). This methodological choice is consistent with and extends existing mixed-methods research in the fields of tourism and information systems (H. Hwang et al., 2017), where SEM is increasingly used to validate complex theoretical models derived from experimental stimuli (S. Kim et al., 2026), thereby providing strong evidence for the sequential transformation from information cues to behavioral intentions.

This study holds significant theoretical implications and practical insights. First, it extends the IAM theoretical framework by supplementing the dual-processing pathways missing in existing IAM research and incorporating a technological perspective on GAI affordance, thereby constructing a model for the adoption of GAI hotel review information. Second, the study reveals how temporal disclosure and review valence, as two dimensions of argument quality, influence consumers’ information adoption through different pathways, finding that the affective pathway consistently exhibits higher effect sizes. Third, it identifies the dimensions of perceived creativity and perceived transparency in GAI affordance and uncovers the moderating role of affordance as a boundary condition. Fourth, it reveals the critical and positive role that psychological distance to the GAI plays in users’ processing of AI-generated review content and clarifies the conditions under which empathy translates into consumer decision-making. This study provides new insights and optimization strategies for theoretical research and practical applications in the field of GAI reviews.

## 2. Literature Review

### 2.1. Information Adoption Model (IAM)

The Information Adoption Model (IAM) proposed by Sussman and Siegal (2003) is primarily used to explain how individuals adopt information and change their behavioral intentions in computer-mediated communication contexts. The model divides information processing into two categories: one based on information quality and the other on source credibility. Both are mediated by perceived usefulness, which in turn influences information adoption decisions.

The IAM has been widely applied to online review adoption in tourism and hospitality. Chong et al. (2018) validated its use in online travel reviews, finding that argument quality and source credibility of eWOM significantly influence travelers’ information adoption through perceived usefulness. Islam et al. (2023) further integrated “information helpfulness” into the IAM framework to develop a new model for online travel review adoption. Salehi-Esfahani et al. (2016) incorporated the technical dimension of website quality and found that review extremity, source credibility, and website information quality collectively influence perceived information helpfulness. With the advancement of GAI technology, IAM has also been applied to the adoption of AI-generated content (AIGC). C. Li et al. (2025) integrated IAM with the Cognition–Motivation–Emotion Framework, extending IAM from static content adoption to dynamic AIGC interaction contexts. J. Wang and Huo (2025) further integrated personality theory with IAM, emphasizing the role of individual differences in AI information adoption. In human–computer interaction research in hospitality and tourism, Pillai and Sivathanu (2020) used TAM as a foundation and integrated human–computer interaction-specific constructs, such as personification, to study tourists’ adoption intentions toward AI chatbots.

A review of IAM theory reveals that scholars have expanded this theoretical model beyond its original conceptual framework to incorporate considerations of the technological dimension. In studies on GAI information adoption, researchers have begun to account for the influence of individual differences; however, they continue to focus primarily on the characteristics of review content as the main influencing factor, overlooking the combined impact of GAI technology and information content characteristics on decision-making outcomes.

#### 2.1.1. Temporal Disclosure and Review Valence

High-quality information can promote users’ information adoption behavior by enhancing perceived usefulness (Sussman & Siegal, 2003). In digital content production involving GAI, the concept of “argument quality” is expanding further. Among these factors, temporal disclosure serves as a key cue for assessing information quality. Temporal cues in information primarily include the time of creation, and temporal disclosure is manifested through the timestamps on comments. Kuchenbecker and Ball (2025) found that temporal disclosure is a core factor in predicting social media engagement, and timely updates to timestamps can significantly enhance the effectiveness of information dissemination. In their study of game reviews, Z. Zhang et al. (2025) found that the disclosure of consumption time influences consumer expectations, which in turn affects how helpful a review is rated; temporal cues influence the assessment of information quality by shaping expectations. In the context of GAI-generated reviews, disclosing the review’s publication time demonstrates the high quality of the GAI-generated content.

Review valence is not merely a sentiment label but a core quality characteristic that influences the persuasiveness of the information. Hong and Park (2012) proposed that the interaction between the affective orientation of online reviews and the type of evidence significantly influences review credibility and product attitudes. Other studies have also pointed out that negative information carries greater weight in consumer decision-making. Researchers (Mudambi & Schuff, 2010; Salehi-Esfahani et al., 2016) found in a study of restaurant reviews that negative reviews are perceived as more useful because negative information is scarcer and more unexpected, and thus more likely to attract cognitive resources. H. Lu et al. (2025) found that AI-generated negative reviews can enhance perceived credibility and improve consumer attitudes toward products, revealing the unique influence mechanism of GAI. Review valence reflects the information quality of GAI-generated content and activates users’ processing of GAI information.

Most existing IAM research focuses on the information quality of online human user reviews, with a lack of adapted information quality assessments in GAI contexts. This study incorporates temporal disclosure and review valence into the dimensions of argument quality, thereby enriching the theoretical implications and practical significance of argument quality.

#### 2.1.2. Psychological Distance to the GAI and to the Hotel

Psychological distance, derived from the Construal Level Theory (CLT) proposed by Trope and Liberman, refers to the degree of proximity that an individual perceives on a psychological level toward a target object or another person, using their own immediate, current experience as a reference point. Essentially, it is a subjective judgment of the degree to which an object is related to the self (Liberman & Trope, 1998).

In online review contexts, consumers perceive a sociopsychological distance from reviewers, and this distance mediates the influence of review characteristics on product evaluations (Hernandez-Ortega, 2018). Ma and Li (2022) found that the perception of psychological distance ultimately influences decision-making by altering judgments of information value. The sources of information on digital platforms are becoming increasingly diverse, with GAI now playing a significant role in the production of online content. The closer consumers perceive their psychological distance to the GAI to be, the more they tend to trust and accept the information it provides. Y. Li et al. (2026), in a study on the use of AI health assistants, confirmed that users’ perceptions of the risks associated with AI technology and their assessments of trust are core factors influencing their willingness to adopt the information. Nguyen et al. (2026), in their study on hotel service robots, also pointed out that the perception of the agency of intelligent technology is a determining factor in the willingness to adopt the technology. Y. Liu and Liu (2026) further verified that students rely on GAI and adopt AI-generated information only when they accept AI as a source of information; the narrowing of psychological distance is reflected in their reliance on GAI. In summary, in online hotel booking scenarios involving GAI, psychological distance to the GAI can be conceptualized as users’ trust in and reliance on GAI.

Unlike psychological distance to the GAI, psychological distance to the hotel manifests as the psychological connection and sense of familiarity consumers form after browsing hotel reviews, reflecting their concrete perceptions of the hotel and their expectations regarding the experience. In hotel and tourism consumption research, psychological distance has been widely used to explain consumers’ information evaluation and decision-making behaviors. Focusing specifically on consumers’ psychological distance to hotels, Jun et al. (2024) confirmed that when psychological distance is greater, exterior photos of hotels receive higher ratings; when psychological distance is closer, interior photos yield better evaluation results. Guillet and Mohammed (2024) further validated the positive predictive role of psychological distance on consumers’ willingness to pay a premium for hotels. S. Zhang et al. (2023) also found that sociopsychological distance significantly influences consumers’ booking behavior in the context of shared accommodations.

#### 2.1.3. Booking Intention

In online hotel bookings, GAI reviews play a central role in assisting users’ booking decisions. Existing research has validated the driving effect of online review characteristics on hotel booking intent, which aligns closely with the core mechanisms of the IAM. He’s (2024) study on Chinese consumers’ hotel booking decisions confirmed that information quality, credibility, and information needs all have a significant positive impact on consumers’ booking intent. The study further refined the impact of review characteristics, finding that reviews revealing user profiles significantly enhance booking intention by improving consumers’ perception of the information source (An et al., 2025). The online word-of-mouth adoption model for hotels developed by Le and Ryu (2023) also shows that source influence and source credibility positively affect hotel booking intention by enhancing trust in word-of-mouth. Shen et al. (2016) noted that, in addition to argument quality and source credibility, group effects also influence consumer purchasing decisions, revealing the complexity of factors affecting purchase intent. S. Kang et al. (2026), through their study of AI-generated travel plans, found that the media richness of AI-generated information influences consumers’ perceptions of trust, which ultimately affects hotel booking intent.

Existing research has validated the association between information content and booking intent in two scenarios—traditional user reviews and AI-generated content—but most studies have focused on single-dimensional perceptual variables and have not yet explored the combined effects of information sources and decision objects.

### 2.2. Elaboration Likelihood Model (ELM)

The Elaboration Likelihood Model (ELM) posits that there are two pathways for individual information processing: when information is highly relevant, individuals tend to analyze the content in depth via the central route; conversely, when information is less relevant, they rely on the peripheral route for affective processing (Petty & Cacioppo, 1984; Schultz et al., 2014). In tourism and hospitality consumption contexts, the explanatory power of the ELM’s dual-pathway framework has been repeatedly validated. Leong et al. (2019), in a study on hotel reservations, found that central and peripheral cues, such as user involvement, review valence, and reviewer expertise, collectively explained 81% of the variance in reservation intention. S. Zhang et al. (2024) further noted that inconsistencies between user-generated and merchant-generated images in the hotel context undermine booking intention through two distinct pathways, perceived risk and negative emotions, which correspond precisely to the central and peripheral processing pathways of the ELM. B. Meng and Choi (2019) also confirmed in tourism service contexts that involvement significantly moderates the strength of the ELM’s dual pathways, with highly involved individuals relying more on the central pathway to form behavioral attitudes.

With the development of generative AI and human–computer interaction technologies, the ELM has been extended to AI-generated content and intelligent interaction scenarios, where its explanatory power has been further validated. G. Tang et al. (2026) explored predictors of user information adoption behavior in human–AI interaction contexts, finding that argument quality and source credibility had significant effects, while technical characteristics had the least impact on the adoption of AI-generated information. Research by Q. Chen et al. (2025) on chatbots showed that recommendation accuracy, as a central cue, and anthropomorphic empathy, as a peripheral cue, both significantly influence users’ willingness to adopt AI-generated information. Research has further confirmed that in GAI-driven shopping recommendation scenarios, central cues such as information quality and peripheral cues such as technical features jointly shape users’ trust in AI and their willingness to adopt it (Chakraborty et al., 2024).

While existing long-term examinations of the ELM have confirmed its explanatory power in the information processing and adoption process, they also suffer from the limitation of being overly broad. Overall, they can only illustrate the information processing process and cannot provide a detailed explanation of complex influence mechanisms; they require integration with other theories to supplement the details before and after information processing.

#### 2.2.1. Cognitive Processing Path

The cognitive processing pathway corresponds to the central pathway in the ELM framework; it is a processing model in which individuals, under conditions of high cognitive involvement, actively mobilize mental resources to systematically analyze information content. Existing research has confirmed that cognitive involvement is a core condition for activating central pathway processing, manifested in consumers drawing upon more cognitive resources and past experiences to process important information. Putrevu and Lord (1994) demonstrated that high cognitive involvement prompts consumers to compare and evaluate the core arguments of a message one by one. Reinhard and Sporer (2008) further validated through experiments that individuals will only engage in deep processing of information and make credibility judgments when task involvement and cognitive capacity are sufficient. In online consumer contexts, Jiang et al. (2010) found that high website interactivity significantly increases users’ levels of cognitive involvement, which in turn positively influences purchase intention. Y. Guo et al. (2023) also confirmed that cognitive involvement is significantly positively correlated with the depth of users’ online information searches and serves as a core driver of in-depth information processing.

When quality characteristics of arguments in reviews—such as temporal disclosure and review valence—trigger consumers’ cognitive involvement, individuals will proactively invest cognitive resources to analyze the hotel information contained in the reviews. This in-depth processing gradually enhances users’ levels of cognitive empathy. Research by S. Wang et al. (2025) on generative AI found that the AI’s responsiveness, logical consistency, and empathetic characteristics enhance users’ product involvement through perceived extrinsic value, with cognitive information processing serving as the core pathway. Meena et al. (2026), in their study on trust in travel chatbots, also pointed out that systematic cognitive processing drives users to form cognitive trust in AI. Cognitive empathy reflects how users understand the content logic of GAI from an information-generation perspective, thereby enabling in-depth cognitive processing of review content. This indicates that users have rationally processed the review content generated by GAI, rather than passively receiving information.

#### 2.2.2. Affective Processing Path

The affective processing route corresponds to the peripheral route in the ELM framework; it is a processing mode in which individuals rapidly form attitudes through heuristic judgments based on peripheral cues such as affective cues and source attributes. Affective involvement is the core variable that activates the peripheral route, leading users to draw on emotional resources when processing content rich in emotional characteristics. Celuch and Slama (1998) found that affective involvement is triggered by affective cues and leads to assimilation processing at the affective level. Hollebeek et al. (2014) also clarified in their study of consumer-brand interactions that emotion is one of the core dimensions of the user interaction experience and can be evoked by peripheral cues without deep cognitive processing. In digital consumption scenarios, X. Xu et al. (2020), in their study of live-streaming e-commerce, confirmed that a host’s attractiveness directly influences consumers’ purchasing behavior through affective arousal.

When affective cues—such as the valence of comments—trigger consumers’ affective involvement, users rapidly undergo affective assimilation toward the information content and perceive the rationality of the GAI’s affective expressions; this process fosters affective empathy in users. J. Han et al. (2022), in their study of short-video tourism scenarios, found that audience involvement directly influences travel intentions through the affective image of the destination. H. Li and Fan (2026), in their research on metaverse retail, further pointed out that peripheral cues such as design aesthetics primarily drive users’ immediate involvement through affective involvement. This affective empathy reflects users’ perception of GAI as a social actor capable of affective expression; they regard its emotional output as a valid reference for decision-making, which helps them form judgments regarding hotel reservations at the emotional level.

### 2.3. GAI Affordance

Affordance theory posits that the impact of technology on individuals is not directly determined by its objective functional attributes, but rather acts upon cognitive judgments and behavioral decisions through users’ perceived possibilities for action and the technology’s characteristics (Nagy & Neff, 2015). Affordance bridges the gap between technological attributes and users’ perceptual responses (M. Wu et al., 2026). GAI affordance refers to the technological characteristics perceived by users during human–computer interaction, including the perceived transparency of GAI information processing and the perceived creativity of GAI information output. Users form perceptual judgments regarding technological affordance while receiving information. Existing research has validated the existence of GAI affordance in multiple contexts, such as environmental education and virtual games, and this technological perception has a significant predictive effect on user engagement and decision-making (Fu et al., 2026; Y. Y. Xu et al., 2026).

#### 2.3.1. Perceived Creativity

Perceived creativity represents users’ overall perception of the originality, expressive richness, and novelty of GAI-generated content, and is what distinguishes GAI from traditional content. In their study on GAI user satisfaction, X. Li and Byun (2026) argued that perceived creativity shapes users’ acceptance of AI-generated content. Yang and Xu (Y. Yang & Xu, 2025) further deconstructed the structure of perceived AI creativity from an audience perspective, pointing out that, in addition to originality and appeal, credibility itself is a core component of perceived creativity.

Existing research has extensively validated the dual impact of perceived creativity on consumers’ content evaluations and decision-making. T. Jung et al. (2025) found in a luxury advertising context that perceived AI creativity simultaneously enhances user trust and perceptions of human-like qualities, thereby strengthening perceptions of the advertising content’s informativeness and novelty. Other studies have also highlighted the boundary effects of perceived creativity. Y. Hwang and Jeong (2026) found that when content is explicitly labeled as AI-generated, excessively high levels of creativity may lead users to question the content’s authenticity, thereby undermining the credibility of the information. In the context of GAI-generated hotel reviews, perceived creativity manifests as characteristics such as the richness, originality, and descriptive novelty of the reviews as perceived by users during information processing. As a core construct of GAI usability, it reflects users’ perceptions of the technology.

#### 2.3.2. Perceived Transparency

Perceived transparency reflects the extent to which users can access information about a GAI’s data sources, generation criteria, and operational mechanisms, and it is a key factor influencing judgments of information reliability. This understanding of the technical logic depends on the technology provider’s disclosure of relevant technical information, but it relies even more on the user’s ability to comprehend the underlying technical logic. As a key indicator of AI technology’s credibility, perceived transparency directly determines users’ trust in AI-generated content and their judgments of its authenticity. Research by M. Jia et al. (2026) on AI-generated review summaries confirms that algorithmic transparency is a core aspect of functional affordance that shapes users’ perceived credibility. Alkhofaily and Noureldin (2026) further elucidated its mechanism of action, showing that the transparency of AI review summaries simultaneously enhances users’ trust in the algorithm and their perceived diagnostic ability, ultimately having a positive effect on purchase intent. Qadri and Moustafa (2026) found in influencer marketing that consumers’ perceived AI transparency positively reinforces the impact of personalized content on purchase intent. Therefore, in the context of GAI-generated hotel reviews, perceived transparency reflects the extent to which users can understand the source and generation rules of the reviews. When users perceive higher transparency—that is, when they better understand the technology—they view the review content as more objective and reliable, thereby reducing concerns about information distortion caused by GAI.

### 2.4. Research Gaps and Integrated Research Model

Existing research has extensively explored the Information Adoption Model (IAM), the Elaboration Likelihood Model (ELM), and the affordance of GAI technology, providing a theoretical foundation for understanding the mechanisms of online review information processing and consumer decision-making. However, in the specific context of GAI-generated hotel reviews, several research gaps remain.

The first concerns the theoretical gap in the IAM. Although the IAM proposed by Sussman and Siegal (2003) provides a foundational framework for research on information adoption, the core logic of its original model is a linear chain: “argument quality/source credibility → perceived usefulness → information adoption.” This model does not distinguish between the cognitive and affective pathways users follow during the information processing process. In the original IAM framework, information processing is simplified into a relatively singular rational evaluation process, and the affective processing mechanisms are completely overlooked. However, in the highly affective information context of GAI-generated comments, users’ processing of affective cues is key to understanding their decision-making behavior.

The second concerns the theoretical limitations of the ELM. Although the ELM provides a framework that distinguishes between cognitive and affective pathways, it is a general-purpose theory of information persuasion and has not been contextualized for GAI technology scenarios (Petty & Cacioppo, 1984; Schultz et al., 2014). In traditional applications of the ELM, researchers often apply it directly to various information-processing scenarios, overlooking the unique challenges posed by artificial intelligence as an information source: users are not only processing the content of the information but also making trust judgments about the technological entity itself. GAI affordances, such as perceived creativity and perceived transparency, are variables not incorporated into the traditional ELM framework.

The third concerns the theoretical limitations of research on GAI affordances. Existing studies on GAI affordances—such as perceived creativity and perceived transparency—largely focus on their direct impact on user attitudes and behavioral intentions while rarely systematically examining how these technological affordances moderate the effects of information content characteristics (M. Jia et al., 2026). In other words, existing research has focused on “what technological affordances influence,” but has rarely explored “how technological affordances alter the way information affects users”—a gap that this study’s moderation analysis aims to fill.

The fourth concerns the limitations in psychological distance research. In hotel and tourism studies, psychological distance has been widely used to explain consumer decision-making; however, existing research often treats psychological distance as a single-level construct, assuming that consumers’ psychological associations directly extend to product evaluations (Guillet & Mohammed, 2024). However, in the context of GAI-generated reviews, consumers are confronted with a dual object: the information generator (GAI) and the object being described (the hotel). Existing theories have failed to clearly distinguish between these two levels of psychological distance and their transmission relationships. The two-level psychological distance framework proposed in this study addresses this theoretical gap.

Based on the analysis of the aforementioned research gaps, this study examines the impact of AI-generated hotel reviews on users’ information adoption and decision-making mechanisms. It employs the IAM framework to break down the “argument quality” dimension into two sub-dimensions: “temporal disclosure” and “review valence,” and the “source credibility” dimension into “perceived transparency” and “perceived creativity.” In exploring the information adoption process, this study identified dual pathways of cognitive and affective processing through the relationships among variables, consistent with the theoretical framework of the Elaboration Likelihood Model (ELM). Consequently, the study incorporates the ELM to explain the information processing mechanisms within the IAM, thereby refining the information processing process: specifically, argument quality influences psychological distance through cognitive and affective processing (cognitive involvement, affective involvement, cognitive empathy, and affective empathy), which ultimately affects booking intention. By incorporating GAI affordance into the source credibility dimension, the study explores how GAI affordance alters the way information influences users. The study clearly distinguishes between psychological distance at the level of the information generator (GAI) and the information subject (hotel), as well as the relationship between them. This integration is not a simple summation of concepts, but rather a systematic construction of consumer information adoption and decision-making mechanisms in the context of GAI reviews.

## 3. Research Model and Hypotheses Development

**Figure 1:**
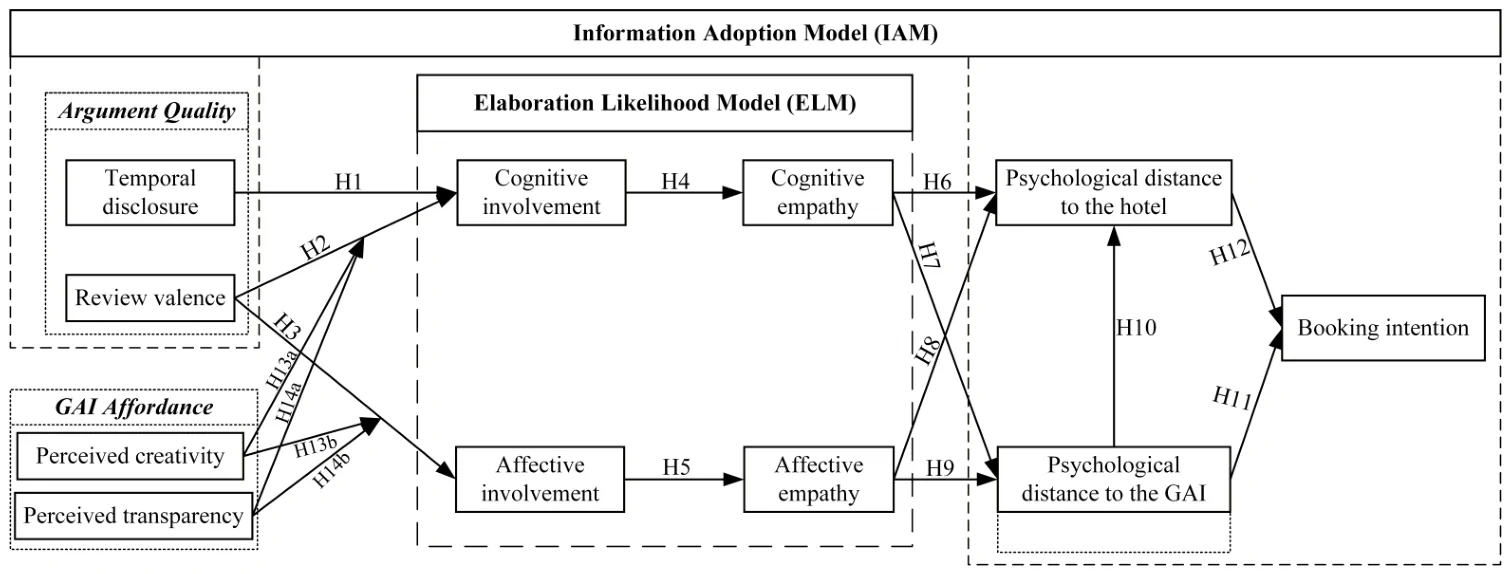
Conceptual model.

Based on the IAM and taking into account the unique characteristics of information processing in artificial intelligence contexts, this study constructs a theoretical model (see Figure 1) to examine how the valence of GAI reviews and the timing of disclosures influence travelers’ booking decisions. This model is based on the IAM framework, incorporates the perspective of GAI affordance, and embeds the dual-process processing pathways of the ELM. It examines how the information quality of GAI (review valence and temporal disclosure) influences psychological distance to GAI and to the hotel through cognitive and affective processing pathways, thereby affecting booking intention.

The integration framework of IAM, ELM, and GAI affordance exhibits specific contextual characteristics when applied to hotel booking scenarios. Specifically, first, the high diagnostic value of hotel reviews means that both the cognitive and affective pathways play important roles. As hotel booking is an experiential form of consumption, consumers rely heavily on others’ evaluations of their experiences. Consequently, this high level of reliance means that both argument quality and GAI affordance have a significant impact on decision-making. Second, GAI reviews hold a unique position in the hotel booking context. Hotel booking platforms (such as TripAdvisor, Booking.com, and Qunar) have widely adopted GAI-generated review summaries to address the information overload caused by the massive volume of user reviews. GAI reviews have become a key information source for consumers during the hotel booking process, meaning this study has a direct real-world application. Third, the experiential nature of hotel consumption makes the psychological distance mechanism particularly important. Hotel reviews are essentially descriptions of consumers’ short-term stay experiences; empathizing with AI-generated hotel reviews essentially involves consumers mentally simulating and previewing their future stay experience. This mental visualization of a distant experience precisely activates the psychological distance mechanism described in the Theory of Interpretive Levels. The sequence of transmission in the two-tiered psychological distance model holds precisely because, in the context of GAI reviews, consumers are confronted with two entities—AI-generated reviews and the hotel itself. They must first trust the source of the information before they can trust the “content” of the review, thereby fostering a sense of closeness.

### 3.1. Temporal Disclosure and Cognitive Involvement

Temporal cues help users comprehensively assess the authenticity and objectivity of GAI information (Luan et al., 2019). In online reviews, temporal disclosure often takes the form of a timestamp indicating when the review was posted. The availability of information, such as the review’s posting date, can enhance users’ deep processing of the information (Hess et al., 2005). Specifically, publication timestamps provide consumers with greater certainty when assessing the credibility of reviews (de Amorim et al., 2022). Information with high credibility increases users’ perceived importance, thereby enhancing cognitive involvement (L. Han et al., 2025). In GAI reviews, the disclosure of the posting time is a key cue for enhancing information richness and credibility (Cheng et al., 2025; B. Li et al., 2021). For GAI reviews that disclose the posting time, users invest more cognitive resources, resulting in stronger cognitive involvement. Therefore, we propose the following hypothesis:

**H1.**  *Temporal disclosure in GAI reviews positively influences users’ cognitive involvement.*

### 3.2. Review Valence and Cognitive Involvement

The valence of a review represents the emotional tone of the information expressed and serves as a key indicator of information quality that determines the depth of consumers’ information processing. Consumers perceive negative statistical reviews as significantly more credible than negative narrative reviews because negative information triggers more detailed processing (Hong & Park, 2012). The perceived usefulness of negative reviews is significantly influenced by factors such as content length because consumers actively invest cognitive resources to evaluate and interpret negative information in depth (Filieri et al., 2019). AI-generated negative reviews can significantly enhance consumers’ perceived credibility because negative content prompts consumers to assess content quality more actively, thereby intensifying cognitive processing efforts (H. Lu et al., 2025). AI-generated negative reviews of hotels typically include information such as service oversights and facility defects, which possess greater decision-making diagnostic value and prompt consumers to mobilize cognitive resources. In contrast, positive reviews generally align with basic expectations of the hotel, contain limited information, and are unlikely to stimulate high-intensity cognitive processing. Therefore, this study proposes the following hypothesis:

**H2.**  *Review valence negatively affects consumers’ cognitive involvement.*

### 3.3. Review Valence and Affective Involvement

According to the ELM’s peripheral pathway, emotional information can trigger an individual’s emotional response through heuristic judgment, in which emotional disposition is a key factor in the affective processing of information. In the mechanism of eWOM influence, comment valence affects consumer attitudes through the mediating role of emotional intensity (Pang et al., 2025). On social media, positive review valence can significantly enhance consumers’ brand attitudes, with emotional resonance serving as a key mediating pathway (Haq et al., 2024). Positive product reviews can effectively alleviate consumers’ negative emotions (Suri et al., 2023). In GAI-generated hotel reviews, positive reviews typically include favorable descriptions of the stay experience, service, and environment, which, through affective processing mechanisms, evoke consumers’ emotional identification and immersion. Therefore, this study proposes the following hypothesis:

**H3.**  *Review valence positively influences consumers’ affective involvement.*

### 3.4. Relationships Between Cognitive Involvement and Cognitive Empathy

Cognitive involvement manifests as an individual’s active mobilization of cognitive resources to conduct a detailed analysis of information, while cognitive empathy manifests as a user’s understanding of the rules governing the generation of information and the perspectives presented. High cognitive involvement prompts consumers to compare and logically deduce the core arguments of the information; this analytical process forms the foundation for cognitive empathy (Putrevu & Lord, 1994). Only when involvement levels are high and cognitive resources are sufficient will consumers engage in systematic information processing and make rational judgments about the credibility of the information (Reinhard & Sporer, 2008). When users are highly involved, they mobilize more cognitive resources to participate in the cognitive processing of information (Yalcin & DiRaola, 2020). In GAI-generated hotel reviews, the higher the level of cognitive involvement among consumers, the more likely they are to actively invest cognitive resources to understand the logic behind the review content. Therefore, this study proposes the following hypothesis:

**H4.**  *Cognitive involvement positively influences consumers’ cognitive empathy.*

### 3.5. Relationships Between Affective Involvement and Affective Empathy

In the peripheral route of ELM, individuals do not need to analyze information in depth; instead, they rapidly trigger affective processing through emotional cues, which is a core condition for the formation of affective empathy. Moreover, when people process information closely related to themselves, they exhibit stronger affective involvement (J. Lee et al., 2024). In addition, emotional cues such as interest can significantly influence audiences’ affective empathy and contribute to the affective processing process (Huang & Liang, 2024). Research on tourism UGC further indicates that emotional content triggers audiences’ affective involvement, driving individuals to engage in heuristic processing and ultimately leading to emotional resonance (Tao et al., 2025). Accordingly, the higher a consumer’s level of affective involvement, the more likely they are to be influenced by the affective expressions and experiential descriptions in reviews, thereby generating an immersive sense of emotional identification. Therefore, this study proposes the following hypothesis:

**H5.**  *Affective involvement positively influences consumers’ affective empathy.*

### 3.6. Relationships Between Cognitive Empathy and Psychological Distance to the GAI and Psychological Distance to the Hotel

In human–computer interaction research, more anthropomorphic GAIs can effectively reduce the psychological distance to GAI, thereby enhancing user satisfaction (C. Liang et al., 2025). When the AI’s identity is disclosed, cognitive empathy plays a key role in interactions between travelers and the AI and influences travelers’ decision-making tendencies (E. J. Kim et al., 2025). Similarly, users’ perceived empathy can also alter their attitudes toward GAIs (J. Lee, 2025). At the mechanistic level, travelers’ perceived empathy toward AIGC reflects their psychological distance to GAI (Thompson et al., 2019). Cognitive empathy can also influence travelers’ relationships with hotels (X. Zhang et al., 2026). Marketers stimulate cognitive empathy by implementing “one-on-one” promotional strategies targeting travelers with limited product experience (Howie et al., 2025). Emerging technologies such as GAI create virtual experiences with varying degrees of realism, which in turn influence travelers’ product perceptions and purchase intentions (J. Wu et al., 2025). Therefore, we propose the following hypotheses:

**H6.**  *Cognitive empathy positively influences psychological distance to the hotel.*

**H7.**  *Cognitive empathy positively influences psychological distance to the GAI.*

### 3.7. Relationships Between Affective Empathy and Psychological Distance to the GAI and Psychological Distance to the Hotel

In human–computer interaction scenarios, the anthropomorphic design of GAI can evoke affective empathy in users, and this empathy effect moderates the extent of its impact on psychological distance (H. Meng et al., 2025). Research on intelligent agents has confirmed that the emotional resonance triggered by anthropomorphic design can effectively bridge the psychological distance to GAI (Lv et al., 2022a). However, if AI-generated spokespersons rely too heavily on anthropomorphic elements such as creativity, they may weaken emotional resonance and increase psychological distance (Yim et al., 2026). In hotel consumption scenarios, information that evokes emotional immersion significantly reduces consumers’ psychological distance to the hotel (Jun et al., 2024). Emotional perceptions at the informational level gradually permeate into product evaluation, systematically influencing consumers’ overall perception of psychological distance to the hotel (Ma & Li, 2022). When consumers experience affective empathy toward GAI reviews, the emotional connection they establish with the information generator transfers to the hotel described in the review, helping consumers construct concrete expectations of their stay and reducing their psychological distance to the hotel. Therefore, this study proposes the following hypotheses:

**H8.**  *Affective empathy positively influences psychological distance to the hotel.*

**H9.**  *Affective empathy positively influences psychological distance to the GAI.*

### 3.8. Relationships Between Psychological Distance to the GAI and Psychological Distance to the Hotel and Booking Intention

The source of information determines its reliability (S. J. Jia et al., 2025b). Travelers’ attitudes toward information sources depend on their psychological distance; the closer the psychological distance, the more likely they are to trust the source, while a greater distance tends to trigger skepticism (X. Li & Sung, 2021). Consumers’ perceptions of a product depend on the information they encounter, and credible cues can effectively aid understanding (J. Guo et al., 2025). When a product has high brand awareness, consumers’ psychological distance from the product is shorter (Marinelli et al., 2025). Influencers (B. Su et al., 2023) and brand ambassadors (Merdin-Uygur & Ozturkcan, 2025) can directly influence consumers’ brand attitudes and purchase intentions. When influencers endorse a product, their followers often make purchasing decisions based on the influencers’ attitudes (Xin et al., 2024). When GAI acts as a brand agent, its creative expressions can reduce the psychological distance between it and travelers, thereby influencing purchase intent (Y. Lu & Zhang, 2025). Existing research has also found that chatbots’ response strategies influence purchase intent by altering the psychological distance between consumers and intelligent agents (H. Meng et al., 2025). Therefore, we propose the following hypotheses:

**H10.**  *Psychological distance to GAI positively influences psychological distance toward the hotel.*

**H11.**  *Psychological distance to GAI positively influences booking intention.*

### 3.9. Psychological Distance to the Hotel and Booking Intention

In consumer and hotel booking contexts, reducing psychological distance often increases the willingness to purchase (Palomino-Tamayo et al., 2022). For example, the image zoom feature on hotel platforms enhances travelers’ familiarity, thereby increasing their purchase intent (Ho et al., 2024). The visual presentation and perceived warmth of hotel advertisements influence travelers’ certainty about the product (Togawa et al., 2023), and this certainty helps reduce psychological distance and fosters positive attitudes toward the product (H. J. Jung et al., 2020). Therefore, reducing the psychological distance between the product and the consumer can effectively stimulate purchase intent. We propose the following hypothesis:

**H12.**  *Psychological distance from a hotel has a positive effect on booking intent.*

### 3.10. Moderating Effects of GAI Affordance

GAI accessibility refers to the technical characteristics perceived by users during human–computer interaction, specifically manifested as perceived transparency and perceived creativity. When receiving information, users form subjective judgments about these aspects of technical accessibility. Users who perceive high accessibility view positive reviews as a sign of AI capability, while those who perceive low accessibility tend to be skeptical of positive reviews. Perceived creativity manifests as technical characteristics perceived by consumers during information processing, such as the richness of review content, the originality of expression, and the novelty of scenario descriptions. Perceived creativity simultaneously influences both affective and cognitive processing pathways, reinforcing the overall impact of informational stimuli (Tie et al., 2024). Highly creative language enhances the uniqueness and distinctiveness of information, amplifying the diagnostic differences in review valence. In e-commerce contexts, the creativity of product descriptions significantly predicts users’ cognitive involvement, reinforcing the driving effect of informational cues on cognitive processing (Mou et al., 2020). Specifically, individuals with different perceptions of the creativity process process information differently; users who perceive high creativity exhibit higher affective involvement (Martucci et al., 2025). Highly creative content expressions possess a stronger emotional appeal and a greater sense of immersion, amplifying the emotional arousal effect of review valence. Therefore, this study proposes the following hypotheses:

**H13a.**  *Perceived creativity positively moderates the relationship between review valence and cognitive involvement; that is, the higher the perceived creativity, the stronger the negative effect of review valence on cognitive involvement.*

**H13b.**  *Perceived creativity positively moderates the relationship between review valence and affective involvement; that is, the higher the perceived creativity, the stronger the positive effect of review valence on affective involvement.*

Perceived transparency is characterized by the extent to which GAI-driven system information is disclosed to users and made accessible (X. Wang & Qiu, 2024). Travelers’ prior perceptions of information sources influence their perception of the system’s transparency, which in turn affects the information processing process (Z. Jia et al., 2025). Low transparency can lead to distrust of GAI (Benlian & Hess, 2011) and weaken travelers’ cognitive involvement (W. Wang & Benbasat, 2016) and affective involvement (L. Liang et al., 2025). High transparency reduces uncertainty, which is a key element of both cognitive and affective involvement (Oldeweme et al., 2021). As a perception of technological affordance, the perceived transparency of GAI-generated reviews influences users’ cognitive and affective involvement across different review valences. Therefore, the following hypotheses are proposed:

**H14a.**  *Perceived transparency positively moderates the relationship between review valence and cognitive involvement; that is, the higher the perceived transparency, the stronger the negative effect of review valence on cognitive involvement.*

**H14b.**  *Perceived transparency positively moderates the relationship between comment valence and cognitive involvement; that is, the higher the perceived transparency, the stronger the negative effect of comment valence on cognitive involvement.*

## 4. Materials and Methods

### 4.1. Mixed-Methods Research Design

This study employed a two-stage mixed-methods design, combining experimental methods with partial least squares structural equation modeling (PLS-SEM), to test the proposed theoretical model. This approach—which integrates experimental methods with structural equation modeling—has been widely used in previous research (S. Kim et al., 2026).

The first phase consisted of the experimental design. We employed a 2 (temporal disclosure: yes vs. no) × 2 (review valence: positive vs. negative) between-subjects experimental design, manipulating the textual content of GAI reviews to simulate a real-world online booking scenario. A pretest was conducted before the main experiment to verify the success of variable manipulation; the specific procedure is described in the experimental steps below. The primary objective of this stage was to ensure, through random assignment of participants, that between-group differences stemmed solely from experimental manipulation, thereby establishing the causal effect of the independent variable on the mediating variable and effectively controlling for interference from extraneous variables.

The second phase consisted of a questionnaire-based PLS-SEM analysis. After participants were exposed to the experimental stimuli, we measured all latent variables in the model via a structured questionnaire, including GAI affordance (perceived transparency and perceived creativity), cognitive and affective involvement, cognitive and affective empathy, psychological distance, and booking intention. Subsequently, this study employed PLS-SEM to analyze the collected data. Compared to traditional analysis of variance (ANOVA), PLS-SEM has the advantage of simultaneously estimating both the measurement model and the structural model, making it particularly suitable for testing the complex model proposed in this study, which includes multiple parallel and chained mediation paths. This method not only verifies the impact of experimental stimuli on proximal psychological states (such as involvement) but also provides deeper insights into how these psychological states ultimately influence the distal behavioral intention (booking intention) through a series of sequential mediating mechanisms (such as empathy and psychological distance), thereby comprehensively mapping the process from informational cues to decision-making behavior.

### 4.2. Measures and Survey Instrument

To ensure the content validity of the scale—that is, the extent to which the items accurately represent the theoretical content of the construct—we identified the latent construct of the initial set of items intended for measurement. This was based on an in-depth review of the literature on consumer behavior, hotel reservations, and generative artificial intelligence. Subsequently, several scales—covering constructs such as perceived creativity, perceived transparency, cognitive involvement, and affective involvement, which had not previously been tested in the context of generative AI-generated hotel reviews—were adapted to our research context to ensure their face validity, i.e., the extent to which respondents perceive the measurement items as appropriate for the target constructs. This adaptation aimed to measure consumers’ perceptions of the quality of AI-generated hotel reviews, their cognitive engagement with the information source, and their willingness to make decisions. All scales used in this study adopted a multidimensional evaluation system adapted from existing literature and employed a 7-point Likert scale (1 = Strongly Disagree, 7 = Strongly Agree).

Specifically, the Perceived Creativity measure drew upon the studies by Casalo et al. (2021) and Q. Li et al. (2025). The original scale for perceived creativity measured the creativity of content posted on Instagram accounts and GAI services; it was adapted in this study to suit the context of AI-generated hotel reviews. For example, the statement “Publications on this Instagram account are revolutionary” was adapted to “The AI review is revolutionary” to align with the research context.

The perceived transparency measure draws on the framework proposed by Yu and Li (2022). The original scale for perceived transparency measures users’ perception of the transparency of AI systems, which aligns extremely well with the context of AI-generated hotel reviews in this study. For example, the statement “I can access a great deal of information explaining how the AI system works” from the original scale corresponds to the measurement of perceived transparency of AI systems in this study.

Cognitive involvement and affective involvement are derived from a previous study (J. M. Kang et al., 2015). The original scale measured users’ cognitive and affective involvement with a mobile location-based retail app; these were adapted in this study to fit the context of AI-generated hotel reviews. For example, “Using this mobile location-based retail app is important” was adapted to “I think AI reviews are important,” and “Using this mobile location-based retail app means a lot to me” was adapted to “I think AI reviews mean a lot to me.”

Cognitive empathy and affective empathy are adapted from the scale developed by Vossen et al. (2015). The original study measured adolescents’ empathy toward others, distinguishing between cognitive and affective dimensions; this study implemented scenario-specific adaptations for AI-generated hotel reviews. For instance, “I can often understand how people are feeling even before they tell me” was revised to “I understood the sentiments conveyed in AI reviews,” and “When a friend is angry, I feel angry too” was modified to “When an AI review expresses dissatisfaction with a product or service, I become angry,” tailored to the research context.

The concepts of “psychological distance to the GAI” and “psychological distance to the hotel” draw on research by H. Chen et al. (2024) and R. Chen and Yan (2025). While prior studies employed scales measuring users’ psychological distance toward virtual live streamers and video creators, this study adapted these metrics specifically for AI-generated hotel reviews. For instance, the statement “I’m familiar with the image of virtual live streamers” was modified to “I’m familiar with AI-generated reviews,” and “I believe my ideas can be noticed by the video creator” was adjusted to “I believe my ideas will be noticed by hotel managers” to align with the research context. Notably, the “psychological distance to the GAI” dimension uses a proximity-oriented scoring system that assesses users’ familiarity with AI reviews, perceived similarity, and psychological affinity; higher scores indicate closer psychological proximity and stronger affinity.

The booking intention metric draws on research by D. J. Kim et al. (2008) and R. Zhou and Tong (2022). The original measurement was derived from consumers’ live-streaming e-commerce purchasing contexts; this study adapted it by tailoring AI-generated hotel reviews to fit the specific research scenario. For instance, “You are willing to consider buying cosmetics while watching the live broadcast” was modified to “I am likely to consider booking this hotel after reading the AI review.”

The questionnaire was initially developed in English, adhering to the translation guidelines established by Brislin (1970). Bilingual experts first translated the scale into Chinese, which was subsequently back-translated into English by another researcher. Following a rigorous translation process, two professors of marketing and communication reviewed the translated questionnaire to ensure its logical coherence and accuracy. All measurement items are detailed in Appendix A (Original Chinese questionnaire is available in the Supplementary File).

### 4.3. Experimental Procedure

This study employs a 2 × 2 between-subjects experimental design (temporal disclosure × review valence) to investigate the impact of GAI on travelers’ decision-making. Authentic AI-generated review content from China’s leading travel platform Qunar.com (X. Guo et al., 2021) was adapted into four distinct experimental stimuli (see Appendix B).

Prior to the main study, a pilot test was conducted via social media platforms to verify the operational validity of the variables “comment valence” and “temporal disclosure.” The pilot study featured four sets of experimental materials and utilized two 7-point scales: “Please rate the degree to which temporal cues are disclosed in the comment (0 = not at all obvious, 7 = very obvious)” and “Please rate the valence of the comment (0 = very negative, 7 = very positive).” Each participant was assigned to only one set of experimental materials, and a total of 107 valid questionnaires were collected.

Among the experimental stimulus materials, those in the temporal disclosure group included the dates of the source reviews (2023–2025), while those in the no-temporal-disclosure group did not include any information regarding the dates of the reviews. Additionally, in the positive review group, the experimental materials praised the hotel’s location, service, room cleanliness, value for money, and facilities. In the negative review group, the experimental materials criticized the hotel’s location, service, room cleanliness, value for money, and facilities.

First, to examine the validity of the temporal disclosure manipulation, the results of an independent samples *t*-test showed that the perceived level of temporal cue disclosure in the time-disclosed group (*N* = 53, *M* = 6.17, *SD* = 0.871) was significantly higher than that in the time-undisclosed group (*N* = 54, *M* = 3.39, *SD* = 1.867), *t*(75.337) = 9.9, *p* < 0.01, *d* = 1.462, 95% *CI* [2.221, 3.340], indicating that the manipulation of the temporal disclosure variable in this study was effective. Next, we examined the validity of the comment valence manipulation. Results from an independent-samples *t*-test showed that the perceived comment valence in the positive comment group (*N* = 54, *M* = 6.22, *SD* = 0.839) was significantly higher than that in the negative comment group (*N* = 53, *M* = 3.19, *SD* = 2.076), *t*(68.297) = 9.875, *p* < 0.001, *d* = 1.578, 95% *CI* [2.421, 3.646], which further confirms that the manipulation of the comment valence variable in this study was effective. The results of the pilot experiment confirmed that the experimental materials effectively distinguished participants along both dimensions of comment valence and temporal disclosure, providing a solid operational foundation for the subsequent formal experiment.

The formal study required participants to be at least 18 years old, with all respondents receiving data confidentiality assurances. The survey was conducted online via Credamo (https://www.credamo.com/#/, accessed on 1 May 2026), a professional Chinese data collection platform with a database exceeding 3 million users. Leveraging its advanced logic control, random sampling, and data verification mechanisms, the platform ensured response authenticity while effectively preventing rushed or arbitrary submissions.

To minimize regional bias, the questionnaire survey covered 28 regions across China, including Beijing, Shanghai, Guangzhou, and Sichuan. IP address restrictions were implemented to prevent duplicate submissions, and a minimum geographic distance of 10 km between participants was enforced to avoid clustering. At the start of the experiment, participants were randomly assigned to one of four condition groups, as shown in Appendix B. Following stimulus exposure, participants completed a questionnaire based on the scales described in Appendix A, measuring all constructs. The questionnaire included one attention check item and two operational check items.

The data collection period for the questionnaire was from July to August 2025. A total of 700 valid questionnaires were collected. After excluding respondents who failed the attention check, 649 final questionnaires were obtained. Demographic analysis revealed that 69.8% of respondents were female and 30.2% were male. Age distribution showed that 43.5% were aged 18–29 and 49.9% were aged 30–39. Among respondents, 75.5% held a bachelor’s degree. According to a report released by Skift Research, women account for 64% of global travelers, drive 82% of travel decisions and more than half of hotel bookings, and are the core audience for online travel content (Thoppil, 2024). Furthermore, the Global Online Travel Market Report indicates that young and middle-aged adults are the main drivers of online hotel spending; in the Chinese market, the 25–40 age group accounts for 68.4% of online hotel booking users, and this group is the core audience for AI-generated travel content (IMARC Group, 2025). The distribution of statistical attributes such as gender and age within the sample aligns with the actual user characteristics in hotel booking scenarios as identified by existing surveys. Furthermore, this study employed Permutation-based Multigroup Analysis (MGA) to test whether there were significant differences in the core research hypotheses across different gender and age groups (Sinkovics et al., 2016); specific results of the sample heterogeneity analysis are presented in Appendix C. Detailed demographic information is presented in Table 1.

To ensure the internal validity of the study, operational validation was conducted through two dichotomous questions: “Did the content you just saw disclose the original review’s publication date?” and “Did the content you just saw recommend booking a hotel?” Subsequently, only responses meeting the experimental conditions (N = 649) were retained for further analysis. This screening process not only validated the operational design’s effectiveness but also ensured subsequent effects could be accurately attributed to the intended stimuli.

## 5. Data Analysis and Results

This study used SPSS 27.0 and SmartPLS 4.1 software for data analysis. First, the reliability, validity, common method bias, and multicollinearity of the measurement model were assessed. Subsequently, the research hypotheses were tested using Partial Least Squares Structural Equation Modeling (PLS-SEM) via the SmartPLS platform. Traditional structural equation modeling (SEM) is parameter-estimation-oriented and has strict statistical requirements, whereas partial least squares structural equation modeling (PLS-SEM) is primarily prediction-oriented and aims to maximize R^2^. The PLS method has fewer sample requirements; data do not need to follow a normal distribution, and it can be used for exploratory and interpretive research without requiring a robust theoretical foundation, making it particularly suitable for exploring the complex models in this study (Kline, 2023). Overall, compared to traditional structural equation modeling, which focuses on optimizing parameter estimates, partial least squares structural equation modeling maximizes predictive power and is therefore more suitable for this study’s exploration of user information adoption and decision-making intentions in GAI scenarios.

### 5.1. Tests for Common Method Bias

Since this study relied on self-reported data, it employed both procedural and statistical adjustments to mitigate common-method bias (CMB). Specifically, to ensure respondent anonymity, the study adopted a randomized sequential questioning approach, distributing questionnaires from different experimental material groups to different respondents at different times.

In addition, common method bias was ruled out through three tests. First, Harman’s one-way ANOVA showed that the first factor explained only 26% of the variance, well below the 50% threshold. Second, following the methods of Podsakoff et al. (2003), we conducted a one-factor confirmatory factor analysis and compared the fit of the baseline model ($\chi^{2}$/df = 2.366, TLI = 0.946, CFI = 0.952, GFI = 0.897, RMSEA = 0.046) with that of the one-factor confirmatory factor analysis ($\chi^{2}$/df = 18.898, TLI = 0.288, CFI = 0.329, GFI = 0.428, RMSEA = 0.166). The results showed that the fit of the single-factor model deteriorated significantly. Third, using the ULMC method, we found that the model fit of the baseline model differed only slightly from that of the model incorporating latent method factors (Δ$\chi^{2}$/df = 0.587, ΔTLI = 0.023, ΔCFI = 0.023, ΔGFI = 0.032, ΔRMSEA = 0.011). Taken together, the results indicate that the common-method bias issue in this study is not significant (Kline, 2023; Richardson et al., 2009).

### 5.2. Assessment of Measurement Model

This study evaluated the measurement model in terms of construct validity, reliability, convergent validity, and discriminant validity. First, the KMO measure of sample adequacy was 0.900, and Bartlett’s sphericity test yielded a significant result ($\chi^{2}$ = 15,242.197, *df* = 595, *p* < 0.001), confirming that the data were suitable for factor analysis (Fan et al., 2021). Second, reliability and convergent validity were assessed. As shown in Table 2, all constructs exhibited good reliability, with Cronbach’s alpha (CA) coefficients ranging from 0.737 to 0.977 and composite reliability (CR) values ranging from 0.834 to 0.983, all exceeding the recommended threshold of 0.7 (Bagozzi & Yi, 1988). The average variance extracted (AVE) for all constructs exceeded 0.5 (ranging from 0.559 to 0.936), fully validating convergent validity (Bagozzi, 1981). All factor loadings were above 0.62, further supporting convergent validity. Table 2 presents the test results for each measurement dimension.

The study also used the Heterotrait–Monotrait ratio (HTMT), a correlation coefficient ratio (Henseler et al., 2015), to assess discriminant validity. As shown in Table 3, all HTMT values were below the conservative threshold of 0.90 (ranging from 0.017 to 0.706), confirming that the constructs differed significantly (Latan, 2018).

Finally, the study tested for multicollinearity by calculating the variance inflation factors (VIFs) for the structural model and the scale items. The VIF values for the structural model ranged from 1.008 to 2.603, while the VIF values for all scale items, excluding the dependent variable, ranged from 1.000 to 4.685. Since all values were below the critical threshold of 5, no multicollinearity issues were found.

### 5.3. Analysis of the Structural Model

**Figure 2:**
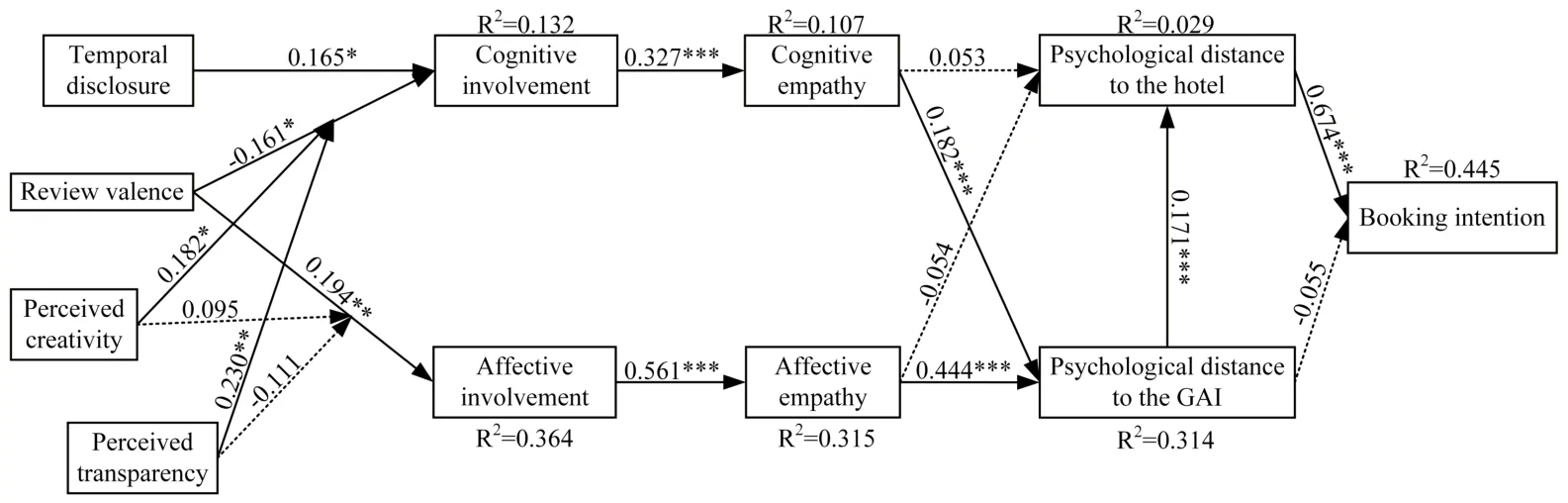
The result of structural equation model. (Explanation: * *p* < 0.05; ** *p* < 0.01; *** *p* < 0.001. Solid arrows indicate supported hypotheses, and dashed arrows indicate unsupported hypotheses).

The study assessed the model’s explanatory power by examining the R^2^ values of endogenous constructs. As shown in Figure 2, the model effectively explains the significant variance in the key variables: cognitive involvement (13.2%), affective involvement (36.4%), cognitive empathy (10.7%), affective empathy (31.5%), psychological distance to GAI (31.4%), psychological distance to the hotel (2.9%), and purchase intention (44.5%). These R^2^ values indicate that the model is explanatory.

The results of the hypothesis tests and the specific data are shown in Figure 2 and Table 4, respectively; most hypotheses were supported. It is worth noting that among the direct effects, H6, H8, and H11 were not supported: the effects of cognitive empathy (β=0.033, t=0.770) and affective empathy (β=−0.054, t=1.190) on psychological distance to the hotel were not statistically significant, and psychological distance to the GAI did not significantly influence booking intention (β=−0.055, t=1.864). This finding suggests that cognitive empathy and affective empathy cannot directly determine users’ psychological distance to the hotel; other factors must also be taken into account (Lin et al., 2023). Another non-significant result indicates that psychological distance to GAI does not directly determine booking intention. This will be discussed in greater depth later in relation to the potential relationships among the variables.

### 5.4. Assessing the Mediation Effects

This study employed the Bootstrap method (with 5000 repeated samples) to test the indirect transmission mechanisms among variables. Given that the direct paths in the main effects tests—H6 “cognitive empathy → psychological distance to the hotel,” H8 “affective empathy → psychological distance to the hotel,” and H11 “psychological distance to the GAI → booking intention” were all rejected, this study excluded the paths containing the aforementioned direct effects from the mediation analysis and focused on testing the chained mediation route in three stages. Detailed specific indirect effects are shown in Table 5.

First, we analyze the three direct paths (H6, H8, and H11) that did not reach statistical significance and discuss them alongside the specific indirect effects. H6 (cognitive empathy → psychological distance to the hotel), H8 (affective empathy → psychological distance to the hotel), and H11 (psychological distance to the GAI → booking intention) all failed to reach statistical significance. Based on the non-significant results for H6 and H8, the study indicates that, regardless of whether it stems from cognitive understanding or affective resonance, consumers’ empathetic response to GAI review content cannot be directly transferred to the hotel described in the reviews. This finding aligns with critical research on the effectiveness of AI empathy. Thomas and Docherty (2026) point out that automated empathy in service contexts may trigger “empathy skepticism” among consumers, leading to alienation and a loss of trust. Perr (2026), drawing on signal theory, argues that when empathy is perceived as AI-generated, its function as a predictive social signal is weakened, making it difficult to convey genuine closeness. Research on voice AI service remediation has also confirmed that AI’s tendency to reduce emotions to quantifiable parameters makes it difficult to convey genuine empathy, thereby undermining consumers’ perception of customer orientation (Carrilho et al., 2025). Existing studies have also found that when empathetic responses are labeled as AI-generated, participants’ perceptions of empathy and support decrease significantly (Rubin et al., 2025). These findings collectively point to a core proposition. In the minds of consumers, AI empathy struggles to translate directly into positive evaluations of the service recipient in the absence of technological credibility.

In the mediation analysis, the study examined the pathway through which empathy mediates via a two-level psychological distance. Given that the direct pathways for H6 and H8 were not supported in the main effects analysis, this study assessed the possibility of a mediation effect among the variables. The core mediation chains “cognitive empathy → psychological distance to the GAI → psychological distance to the hotel” and “affective empathy → psychological distance to the GAI → psychological distance to the hotel” were tested sequentially. The results showed that the effect sizes for “CE → AIPD → HPD” and “AE → AIPD → HPD” were β=0.031(p<0.01) and β=0.076(p<0.001), respectively, with both mediation effects reaching statistical significance. This indicates that psychological distance to the GAI fully mediates the relationships between “cognitive empathy and psychological distance to the hotel” and between “affective empathy and psychological distance to the hotel.” This result supports some of the conclusions from the main effects: cognitive empathy must reduce consumers’ psychological distance to the GAI to subsequently reduce their psychological distance to the hotel; similarly, affective empathy must also reduce psychological distance to the GAI to subsequently influence psychological distance to the hotel. Research on empathy toward AI agents provides a detailed explanation: cognitive empathy primarily influences users’ willingness to engage through perceived novelty, while affective empathy operates through perceived warmth; the dual mediation of warmth and novelty jointly shapes the impact of empathy on behavioral intent (S. Zhang et al., 2025). This finding complements the conclusions of the present study. Together with existing research, this study demonstrates that the influence of empathy is not nonexistent; rather, it must be transmitted to the decision-making object via a perceptual evaluation of the technological agent. In the absence of this mediating bridge, the empathy effect cannot manifest.

The non-significant result in H11 further reveals the hierarchical relationship between trust in technology and consumer decision-making. Previous research has shown that personification enhances users’ evaluations of AI assistants by reducing psychological distance. Still, this effect is primarily manifested at the level of attitudes toward the technological entity (X. Li & Sung, 2021). Empirical studies confirm that consumers perceive greater psychological distance when interacting with AI recommendation agents, and that this distance determines the effectiveness of persuasive messages; however, these studies also suggest that the impact of psychological distance on behavioral intentions is mediated by information processing pathways (Ahn et al., 2021). Existing research comparing the effects of human and AI agent services on customer learning and loyalty has found that the indirect effect of AI interactivity on loyalty requires full mediation by customer learning and immersive experiences (Chatterjee et al., 2025). These studies indicate that consumers’ trust in and affinity for information sources merely form the basis for decision-making; they must be transformed into actual consumption intentions through positive expectations regarding the decision object.

In the mediation analysis, this study verified that “psychological distance to the GAI” influences “booking intention” through the mediating effect of “psychological distance to the hotel,” with β=0.115(p<0.001). The results also indicate that “psychological distance to the hotel” fully mediates the relationship between “psychological distance to the GAI” and booking intention. “Psychological distance to the GAI” cannot directly influence booking intention; rather, it must first influence “psychological distance to the hotel” before ultimately affecting booking intention. This finding suggests that consumers ultimately book the hotel itself, rather than the GAI as an information tool; this fundamental decision-making logic has been clearly statistically validated in this study.

Second, the study examined the stage-specific mediation of temporal disclosure and review valence through dual-path processing. In the cognitive path, both temporal disclosure and review valence indirectly and significantly influenced cognitive empathy through cognitive involvement, with effect sizes of β=0.054(p<0.05) and β=−0.053(p<0.05), respectively, reflecting the central path characteristics of the ELM. Temporal disclosure increased cognitive involvement, while negative reviews drove deep cognitive processing through their diagnostic advantage, ultimately promoting cognitive empathy. In the affective pathway, comment valence indirectly and positively influenced affective empathy through affective involvement, with an effect size of β=0.109(p<0.01), reflecting the characteristics of a peripheral pathway. Emotional cues can rapidly evoke users’ emotional resonance. In comparison, the mediating effect of comment valence via the affective pathway was significantly stronger than that via the cognitive pathway.

Third, the results of the chained mediation analysis revealed significant differences in the effects across different pathways. Specifically, the chained mediation effect in the affective processing pathway (review valence → affective involvement → affective empathy → psychological distance to GAI → psychological distance to the hotel → booking intention) was significant (β=0.006,p<0.05), whereas neither of the two chained mediation effects under the cognitive processing pathway reached the level of significance.

Fourth, we turn to the results of the path analysis on the mechanism of temporal disclosure. The statistical results show that the distal indirect effect of temporal disclosure on booking intention via the full chain of pathways was not statistically significant. The findings suggest that the distal influence of temporal cues on final decision-making intent may be subject to effect attenuation, or may require shorter psychological transmission chains and moderation by other boundary conditions. However, the first half of the mediation path—“temporal disclosure → cognitive involvement → cognitive empathy”—reached statistical significance. This finding holds significant theoretical implications: it confirms that temporal disclosure, as a form of technological affordance cue, can effectively stimulate consumers’ cognitive involvement first, thereby prompting them to develop cognitive empathy from the perspective of information-generation logic. Although this cognitive chain did not fully translate into a significant change in booking intention, it clearly illustrates the central processing trajectory when users process GAI information. This provides procedural evidence for the application of ELM theory in the context of AI-generated hotel reviews, demonstrating that even simple temporal disclosure can precisely trigger and guide users into a mode of deep cognitive processing.

Fifth, we turn to the low R^2^ value (2.9%) of the “psychological distance to the hotel” variable in the model structure. In this model, “psychological distance to the hotel” occupies the final link in the mediation chain. Its effect primarily stems from the transmission of “psychological distance to the GAI” (H10: β=0.171, p<0.001), rather than being directly explained by the empathy variables. In a chained mediation model, explanatory power gradually diminishes as the effect is transmitted sequentially through preceding variables. Research on AI service remediation has found that highly empathetic AI responses enhance customers’ intention to continue using the service through the chained mediation of psychological distance and trust, indicating that in AI–human interaction contexts, the terminal mediating effect of psychological distance is often constrained by the explanatory power of preceding variables (Lv et al., 2022b). A similar pattern exists in research on travel cancellation behavior: the influence of psychological distance on behavioral outcomes must be mediated through cognitive and affective processes, resulting in a significant weakening of the direct predictive power of the terminal variable (H. Lee et al., 2021). This structural characteristic objectively limits the upper bound of the explanatory power of “psychological distance to the hotel.” Still, it also suggests that future research could enhance explanatory power by incorporating a more diverse set of predictor variables. In summary, the low explanatory power of “psychological distance to the hotel” does not undermine the validity of this study’s core findings; rather, it reveals the complexity of the construct’s formation mechanism. Other potential factors contributing to the model’s low explanatory power will be discussed in the Section 6.3.

### 5.5. Assessing the Moderating Roles of GAI Affordance

**Figure 3:**
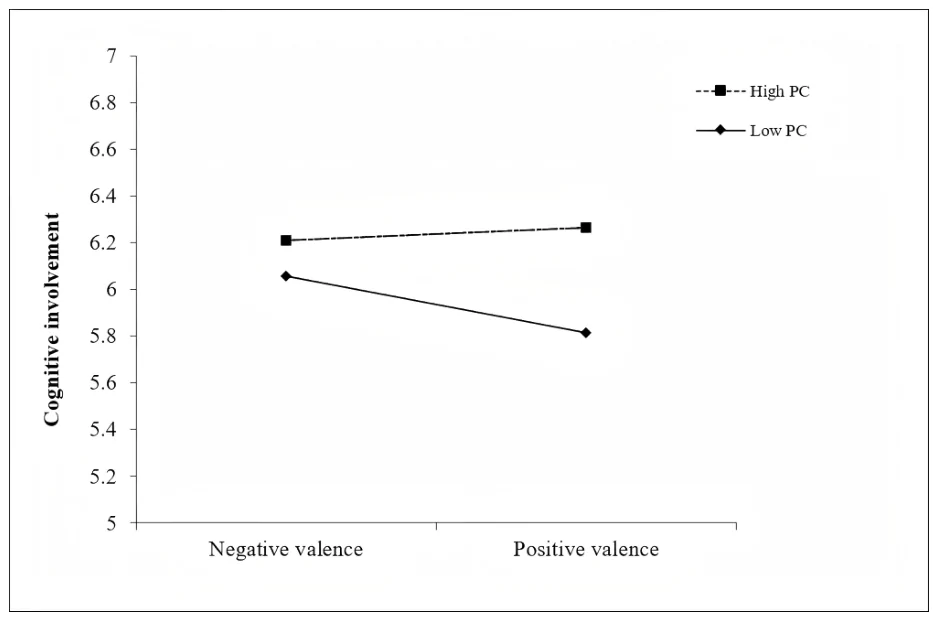
Moderating effects of perceived creativity.

**Figure 4:**
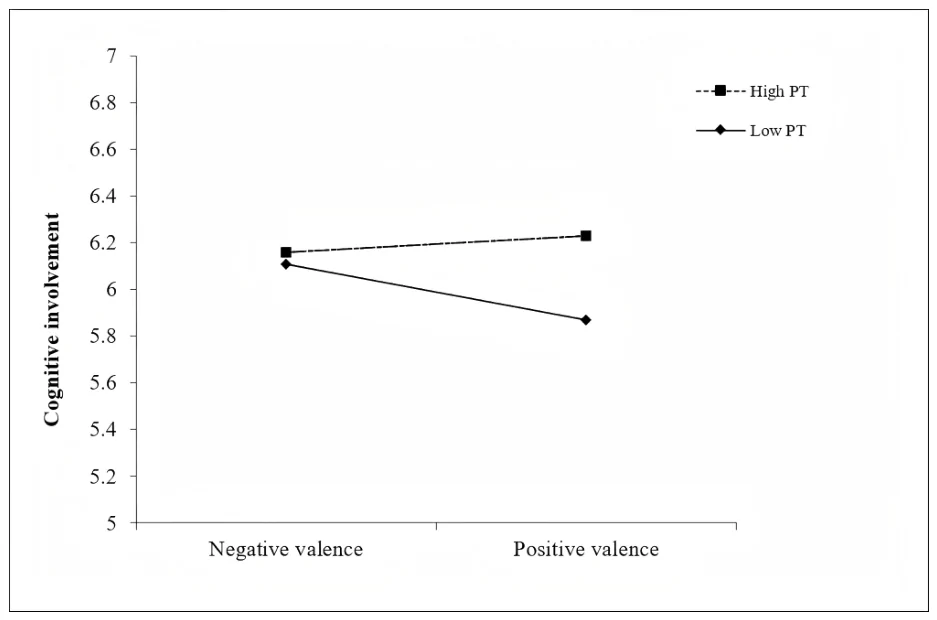
Moderating effects of perceived transparency.

This study included perceived transparency (PT) and perceived creativity (PC) regarding GAI affordance as moderating variables in the analysis and illustrated their moderating effects by plotting interaction effect diagrams (see Figure 3 and Figure 4).

The test results show that the moderating effects of perceived creativity and perceived transparency exhibit consistent path characteristics: both exert a significant positive moderating effect on the relationship between comment valence and cognitive involvement, while they have no significant moderating effect on the path between comment valence and affective involvement. Specifically, the positive moderating effect of perceived creativity on the relationship between comment valence and cognitive involvement was significant (β=0.182, t=2.071,p<0.05), supporting H13a; its moderating effect on the relationship between review valence and affective involvement did not reach the level of significance (β=0.095, t=1.128,p>0.05), and H13b was not supported. As shown in the interaction effect plot, due to the negative main effect of review valence itself, consumers generally exhibited higher levels of cognitive involvement under negative reviews. When the review valence shifted from negative to positive, the level of cognitive involvement among consumers with low perceived creativity declined significantly, while that of consumers with high perceived creativity remained at a relatively high level. This indicates that perceived creativity can effectively mitigate the negative effect of review valence on cognitive involvement; the stronger consumers’ perception of the creativity of GAI-generated information, the weaker the inhibitory effect of positive reviews on cognitive processing effort.

Similarly, the positive moderating effect of perceived transparency on the relationship between review valence and cognitive involvement was significant (β=0.230, t=2.652, p<0.01), confirming H14a; however, its moderating effect on the relationship between review valence and affective involvement was not significant (β=−0.111, t=1.304, p>0.05), and H14b was not supported. Judging from the trends in the interaction effect plot, in the negative review scenario, the difference in cognitive involvement levels between the high-perceived-transparency group and the low-perceived-transparency group was small; upon entering the positive review scenario, the cognitive involvement level of the low-perceived-transparency group decreased significantly, while that of the high-perceived-transparency group fluctuated very little, and the gap between the two groups gradually widened. This result indicates that perceived transparency can also weaken the negative association between review valence and cognitive involvement; the higher the perceived transparency of GAI technology, the more likely consumers are to maintain a high level of cognitive involvement when faced with positive reviews.

Perceived creativity and perceived transparency both exerted significant moderating effects on the cognitive involvement pathway while having no significant impact on the affective involvement pathway. This differential moderation pattern contrasts with findings in previous studies suggesting that perceived affordance simultaneously enhances both cognitive and affective pathways (Feng & Liu, 2025; J. Li et al., 2026), indicating that the role of GAI-affordance exhibits clear pathway selectivity. To understand this discrepancy, it is necessary to conduct a theoretical analysis of the essential attributes of affordance perception and its relationship with different information processing pathways.

From the perspective of the informational attributes of affordance perception, both perceived creativity and perceived transparency are rational cues that require cognitive evaluation, rather than emotional cues that directly elicit emotional responses. Y. Yang and Xu (2025) point out that, in addition to originality and appeal, credibility itself is a core component of creativity perception; this implies that creativity perception inherently involves rational judgment regarding the reliability of the information source. Similarly, perceived transparency reflects the extent to which users can understand the data sources, generation criteria, and operational mechanisms of GAI (W. Jia et al., 2026; Yu & Li, 2022); in essence, it represents the cognitive accessibility of the underlying technological logic. When consumers encounter positive reviews, high levels of creativity and transparency can provide additional diagnostic information, thereby compensating for the relative lack of information in positive reviews and sustaining users’ cognitive engagement. This logic is intrinsically consistent with the findings of S. Zhang et al. (2025), who demonstrated that AI transparency enhances users’ cognitive engagement by increasing the diagnostic value of information.

In terms of information-processing pathways, affective involvement is primarily triggered automatically by emotional content in reviews—such as emotional vocabulary and descriptions of experiences—and falls under the category of heuristic processing that relies on peripheral cues within the ELM framework (Petty & Cacioppo, 1986). This emotional arousal process is characterized by automation and low cognitive load, and is not easily influenced by consumers’ perceptions of GAI’s technical capabilities. J. Tang (2026) found that the impact of technological affordance on flow experiences and willingness to communicate is significantly moderated by learner agency, suggesting that the role of technological perception in the affective pathway depends on the extent to which individuals actively mobilize psychological resources. Research on GAI users’ willingness to disclose also indicates that anthropomorphic interaction affordances, such as social presence, primarily exert their effects through the affective pathway, whereas content-generation affordances mainly influence the cognitive level; affordance types are differentially matched to processing pathways (T. Zhou & Wu, 2025). In other words, regardless of how creative or transparent consumers perceive GAI to be, descriptions of pleasant experiences in positive reviews can still effectively evoke their affective resonance; similarly, expressions of negative emotions in negative reviews can directly trigger affective processing. The level of GAI affordances cannot fundamentally alter this basic pattern of affective response.

Furthermore, from the perspective of affordance realization, Shao et al. (2025) found in their study of AI voice assistants that the impact of technological affordance on users’ cognition is moderated by boundary conditions such as frequency of use, and that affordance realization does not occur unconditionally. W. Lu et al. (2025) further verified that the transformation from affordance perception to affective responses requires cognitive mediation. When consumers process GAI reviews, whether perceiving creativity or transparency, the first response triggered is a cognitive evaluation of argument quality and source credibility, rather than a direct affective response. This cognitive-first processing sequence determines that the moderating effects of GAI affordance are more likely to manifest along cognitive pathways. Z. Zhou et al. (2026) point out that the same technological feature may simultaneously produce both promotional and inhibitory effects, which offers an alternative perspective on the limited moderating effects of perceived creativity and perceived transparency in this study: the impact of technological affordance on user psychology is not a simple linear enhancement. Still, it may produce threshold effects or even negative effects under specific conditions.

The findings of this study indicate that the influence of GAI affordance has clear boundaries, with its moderating effects primarily concentrated at the cognitive level—which requires rational analysis—rather than at the level of affective responses. This conclusion enriches the application of affordance theory in the context of GAI reviews and responds to calls from existing research to identify patterns of AI affordance in specific decision-making contexts (Bao et al., 2023). The study’s findings also provide theoretical support for understanding how user preferences in AI-assisted travel planning are moderated by contextual factors (Hrankai & Mak, 2026): the effects of GAI affordance are not universal. Still, they are highly dependent on task type, information processing stage, and the user’s cognitive state.

## 6. Discussion

GAI has demonstrated significant application potential in areas such as information integration and hotel reservations, effectively assisting users in obtaining and understanding relevant product or service information. However, in the context of GAI-generated hotel reviews, there remains a lack of empirical research on how review valence and timing of disclosure influence booking decisions. To address this research gap, this study designed a scenario-based online experiment involving 649 consumers. The experiment systematically examined how the temporal disclosure and valence of GAI reviews, as well as GAI affordance, collectively influence booking decisions. The theoretical contributions and practical implications of this study are elaborated below.

### 6.1. Theoretical Contribution

First, this study constructed a GAI review information adoption model by integrating the affordance dimensions of IAM, ELM, and GAI, thereby expanding the theoretical framework of IAM to explain the mechanisms by which users process AI-generated content. Although existing IAM research has been extended to the field of AI-generated content adoption, it has not yet fully incorporated the affordance characteristics of AI technology (C. Li et al., 2025; J. Wang & Huo, 2025). While research based on the ELM can explain the dual-path mechanism of information processing, it can only depict the information processing process and cannot provide a detailed explanation of complex influence mechanisms (Schultz et al., 2014). This study incorporates temporal disclosure and review valence as expanded dimensions of argument quality into the IAM framework, treats cognitive empathy and affective empathy as outcome variables of the ELM dual-pathway model, and introduces GAI affordance as a moderating variable to construct a model for the adoption of GAI hotel review information. This integration provides an expandable analytical framework for future research on GAI information adoption. At the theoretical level, this model retains the IAM’s core focus on the relationship between information characteristics and adoption decisions, enhances the explanatory power of the process by incorporating the dual pathways of the ELM, and captures the uniqueness of AI technology by including GAI affordance. It effectively integrates these three theoretical perspectives, providing a more detailed explanation of users’ information processing and adoption processes regarding AI-generated reviews.

Second, this study reveals how temporal disclosure and review valence—as two dimensions of argument quality—influence information adoption through different pathways, and finds that the effect size of the affective pathway is larger than that of the cognitive pathway. The effects of the two pathways in the ELM are highly context-dependent. Research on social media user comments has found that cognitive language operates through the central pathway, while emotional words operate through the peripheral pathway, confirming the coexistence of both pathways in enhancing consumer involvement (ShabbirHusain et al., 2024). In the context of AI-generated digital content, the dominant dual-pathway pattern exhibits more complex characteristics. Existing research has found that the cognitive pathway for processing argument quality is more effective in AI interaction scenarios (G. Tang et al., 2026). Hsu et al. (2026) compared information processing from human and AI sources and found that human recommendations activate both pathways simultaneously, whereas AI recommendations rely more heavily on the central pathway. These studies collectively point to the dominant role of the cognitive pathway in AI information processing.

This study identifies and substantiates temporal disclosure as a new contextual dimension of argument quality in AI-generated reviews and reveals its unique mechanism of influencing cognitive empathy through the mediating role of cognitive involvement. Human reviews on digital platforms typically display the posting time by default. A large body of research confirms that temporal cues serve as a core basis for consumers to judge the timeliness, relevance, and diagnostic value of reviews (Jin et al., 2014; Y. Yang et al., 2018), with their influence even surpassing that of “helpful” votes (S. Lu et al., 2018). Not only is this the primary factor determining review readership, but it also influences persuasiveness by aligning with consumers’ purchase timeframes (J. Lee, 2013). However, AI-generated review summaries on current mainstream platforms generally omit this information. Therefore, “whether to disclose” is not an inherent attribute but rather a strategic choice platforms face when deploying GAI. This study finds that restoring temporal disclosure in AI reviews significantly enhances consumers’ cognitive involvement, indicating that temporal disclosure compensates for the lack of information richness and credibility in AI content caused by decontextualization. This resonates with research showing that goal disclosure enhances commitment (L. Su et al., 2022), establishing temporal disclosure as a new dimension of argument quality within the IAM framework in AIGC contexts and addressing gaps in existing research regarding the informational characteristics of AI scenarios.

This study also revealed the psychological mediation pathway of temporal disclosure. On the one hand, the distal indirect effect of temporal disclosure on booking intention via a full chain pathway did not reach statistical significance. Therefore, this study’s conclusion is limited to the finding that temporal disclosure exerts a robust driving effect at the proximal stage of cognitive processing. The study found that temporal disclosure first stimulates cognitive involvement (H1), which in turn promotes cognitive empathy (H7). This mediating effect indicates that temporal cues first prompt consumers to allocate more cognitive resources to systematic processing, subsequently driving them to understand GAI from the perspective of information-generating logic. This finding provides clear procedural evidence for the operation of the ELM central pathway in AI information processing, demonstrating that even simple choices in technical presentation can precisely trigger deep cognitive processing.

However, this study found that the indirect effect of comment valence on affective empathy via affective involvement was more significant than the indirect effect via cognitive involvement on cognitive empathy; in the full chain mediation analysis, only the full chain of affective processing reached statistical significance. This result not only validates the findings of Shi et al. (2026), who demonstrated that negative emotions act as peripheral cues to accelerate decision-making, but it also extends the discussion of this finding to the GAI review scenario, where the emotional orientation of GAI reviews can still serve as a peripheral cue to rapidly trigger users’ heuristic processing. The study confirms the coexistence of dual pathways in AI content and expands upon the dynamic perspective of cognitive and emotional complementarity proposed by Teubner et al. (2024). This study further identified that the affective pathway plays a stronger role than the cognitive pathway in the context of GAI hotel reviews, thereby enhancing the theoretical explanation of ELM for research on GAI applications in specific scenarios.

Third, this study reveals the buffering moderating effect of GAI affordance and identifies the moderating patterns of perceived creativity and perceived transparency as boundary conditions. Previous studies have separately validated the direct effects of perceived technological creativity (Sun et al., 2024) and perceived transparency (X. Wang & Qiu, 2024) on user trust and behavioral intent. However, few studies have systematically examined how these aspects of technological affordance moderate the effects of information content characteristics. The findings of this study advance the application of affordance theory in the context of GAI reviews; the moderating effects of perceived creativity and perceived transparency are not, as widely assumed in previous research, active across both cognitive and affective pathways (Tie et al., 2024), but are instead concentrated on the cognitive pathway.

Additionally, the study revealed the buffering nature of these moderating effects. Analysis of the data indicates that high GAI affordance can weaken the inhibitory effect of positive reviews on cognitive involvement. This result echoes the findings of Y. Hwang and Jeong (2026) regarding the potential for AI-generated content labels to undermine credibility, suggesting that consumers’ doubts about the authenticity of AI-generated positive reviews are resilient and cannot be fully dispelled by enhancing perceived creativity or transparency alone. This also corroborates Park’s (H. E. G. Park, 2024) view that generative AI possesses both affordances and constraints.

Furthermore, the findings of this study regarding the moderating effect of perceived transparency engage in a theoretical dialogue with the studies by Bian et al. (2025) and S. Wang et al. (2026), which suggest that transparency indirectly influences public attitudes through perceived trust. This study indicates that the role of transparency is most pronounced during the cognitive stage of information processing, providing procedural evidence for the notion proposed by K. Park and Yoon (2025) that “transparency is a conduit for trust rather than a prism”: transparency builds trust because it first facilitates users’ systematic cognitive processing of information, rather than simply eliciting affective favorability.

Fourth, the study reveals the critical and positive role that psychological distance to GAI plays in users’ processing of AI-generated reviews, clarifying the conditions under which empathy translates into consumer decisions. Existing research on the impact of information processing on consumer decisions posits that empathy can directly influence judgments of psychological distance toward a product. In the context of hotel AI service remediation, highly empathetic AI responses can increase users’ willingness to continue using the service by reducing psychological distance (Lv et al., 2022b). Yim et al. (2026), however, provide counterevidence, showing that low empathy elicited by AI spokespersons widens consumers’ psychological distance. Most of these studies treat psychological distance as a single-level construct. They assume that the psychological connection generated by empathy directly extends to the evaluation of products or services. They fail to distinguish between the dual-level distance structure—comprising psychological distance from the information source and psychological distance from the decision object—and they do not clarify the boundaries of empathy’s role in human–computer information interaction.

Regarding empathy, this study found that neither cognitive empathy generated via the central pathway nor affective empathy generated via the peripheral pathway can directly influence consumers’ psychological distance to the hotel (H6 and H8 are not supported). Instead, they first reduce the psychological distance to the GAI—the information-generating entity—and then indirectly influence perceptions of the hotel through trust transfer. This finding aligns with Alsaid et al.’s (2026) concept of the shift in psychological distance from the technological agent to the service object, confirming that in an environment where information is generated by GAI, consumer empathy first anchors to the technological agent for distance assessment and then spreads to the decision object, rather than acting directly on the product itself.

This study also found that consumers’ psychological distance to GAI cannot directly influence their willingness to book a hotel (H11 is not supported); it must be fully mediated through psychological distance to the hotel to exert an effect. This indicates that, as an information-generating tool, the source-level psychological connection established by GAI can only endorse information credibility; it cannot directly translate into final product consumption decisions. This finding provides cross-scenario corroboration with the research findings of M. Wu et al. (2026) on GAI-enabled public service scenarios. It also challenges the widespread perception in the field of human–computer interaction that trust in technology directly drives behavior. The finding further resonates with the logic of trust transfer in online reviews and tourism marketing, where perceptions of credibility at the source level must pass through perceptions at the object level—such as the destination’s image—to effectively drive travel intent (S. Chen et al., 2026). This clarifies that, in scenarios where GAI generates hotel reviews, psychological distance at the technological level has clear boundaries of influence; its effects must be grounded in value judgments regarding the product itself to extend to consumption decisions.

### 6.2. Practical Implications

The findings of this study offer differentiated practical implications for key stakeholders in the hotel booking ecosystem.

First, hotel operators should strive to optimize the affective expressions in their reviews. The study found that the affective processing pathway exerts a significantly stronger influence on decision-making regarding GAI reviews than the cognitive pathway. This implies that the affective tone and experiential details retained in GAI reviews play a more prominent role in driving booking intention than purely objective descriptions. Therefore, businesses should actively encourage guests to incorporate specific descriptions of emotional experiences into their reviews, providing richer material for GAI summaries.

Second, platform operators should optimize presentation design and guidance for trust transfer. Currently, the absence of timestamps in GAI content within online hotel booking and e-commerce scenarios constitutes a shortcoming in argument quality, which has become a major obstacle to building trust between humans and machines (Guan & Lin, 2025; Kirpalani, 2025; Y. Zhang et al., 2025). Research has found that the temporal disclosure of review information has a significant positive impact on cognitive involvement; platforms should clearly label the time of reviews within the GAI review interface to stimulate consumers’ cognitive processing engagement. Regarding trust transfer, studies have shown that psychological distance to the GAI does not directly influence booking intention but exerts its effect only through the full mediation of psychological distance to the hotel. Platforms should embed interactive buttons such as “View Hotel Details” following the GAI review display area to guide users through the psychological transition from a trusted information source to a familiar information object.

Third, algorithm developers should balance argument quality, affective expression, and transparency. The positive effects of the affective pathway suggest that algorithms should not merely pursue objectivity and neutrality but should moderately retain affective vocabulary and experiential descriptions in reviews to enhance users’ affective processing. Perceptual creativity and transparency both moderate only the cognitive pathway; designs aimed at enhancing affordance primarily serve scenarios requiring rational evaluation, while activating the affective pathway still relies on the rich affective material found in original reviews. The focus of optimization is not to replace the affective pathway with affordance, but to use affordance to underpin the credibility foundation for affective transmission.

### 6.3. Limitations and Future Research

This study has several limitations that may provide avenues for future research.

First, regarding temporal disclosure, this study only considered whether a comment’s posting time was disclosed, neglecting the impact of detailed temporal cues on user involvement. Research on temporal disclosure is still in its preliminary stages; future studies could explore detailed temporal cues, such as timeliness and richness.

Second, this study focuses on the impact of AI-generated hotel reviews on user decision-making and does not include a control group of human-generated reviews; this limits, to some degree, the extent to which the study’s findings can be attributed to the unique characteristics of GAI. However, it should be noted that the AI review material used in this study is essentially a distillation and summary of a large volume of original user reviews, and its content—such as descriptions of the guest experience and service evaluations—is derived directly from the actual feedback of real consumers who stayed at the hotels. Therefore, the “review valence” and “temporal disclosure” effects observed in this study reflect, to some extent, the role that AI-integrated user review information plays in influencing consumer decision-making. Future research could design more systematic comparative experiments by juxtaposing original human reviews with AI-generated review summaries to directly compare whether there are significant differences in consumers’ processing and adoption mechanisms for these two types of review sources, thereby more precisely isolating the unique psychological effects resulting from AI’s role as an information integrator.

Third, this study could only define the stronger role of affective processing pathways in terms of statistical significance and variance explained. Future research could employ process-tracking methods such as eye tracking, EEG, or reaction time measurements to directly observe users’ cognitive and affective responses when processing GAI reviews, thereby providing more direct evidence of the processing mechanisms.

Fourth, the demographic characteristics of the sample show a certain concentration in terms of age and gender; this distribution aligns closely with the core user profile of online travel booking platforms. However, “aligning with the industry’s user profile” does not equate to “covering all consumer groups.” The proportions of middle-aged and older adults (aged 50 and above) and male consumers in the sample were relatively low, so caution is warranted when generalizing the study’s conclusions to these groups. Future research could use quota sampling or supplementary surveys targeting specific populations to test the robustness of this model across different demographic groups. Future research could also conduct replication studies in other cultural contexts to test the model’s cross-cultural applicability.

Fifth, we turn to the low R^2^ value for “psychological distance to the hotel” in the model structure. Given the multidimensional nature of psychological distance and the limitations of single cues, psychological distance, derived from the theory of explanatory levels, is essentially a multidimensional construct encompassing temporal distance, spatial distance, social distance, and hypothetical distance. In the field of tourism and hospitality research, scholars have repeatedly validated the differentiated effects of these dimensions on consumer judgments and decisions. Analysis of large-scale review data from TripAdvisor and Booking.com revealed that temporal and spatial distances have a positive, direct impact on review valence (Stamolampros & Korfiatis, 2018). In a study of restaurant reviews, H. Li et al. (2021) found that temporal distance has a positive effect on review consistency, with the valence of the consumption experience and the type of review platform serving as boundary conditions. Harris and Istanbulluoglu (2025) further revealed how the four dimensions of psychological distance collectively shape review valence, indicating that each dimension of psychological distance exerts independent and interactive effects on the generation and evaluation of review information. These studies indicate that consumers’ judgments of “psychological distance to the hotel” result from the combined effects of multiple dimensions of distance. In contrast, the experimental stimuli in this study focused solely on two types of textual information—review valence and temporal disclosure—and neglected information inputs from multiple dimensions such as spatial distance and social distance. In summary, the low explanatory power of “psychological distance to the hotel” does not undermine the validity of this study’s core findings; rather, it highlights the complexity of the construct’s formation mechanism. It also points the way for future research: incorporating multimodal information cues and contextual factors to enhance the model’s explanatory power regarding this key mediating variable.

## 7. Conclusions

This study aims to reveal how hotel reviews generated by generative artificial intelligence (GAI) influence travelers’ information adoption and booking decisions through dual cognitive and affective processing pathways, mediated by temporal disclosure and review valence, and examines the moderating role of GAI affordance. The study found that temporal disclosure and review valence, as two dimensions of argument quality, activate dual-pathway information processing, with the affective pathway exhibiting a significantly stronger effect than the cognitive pathway. GAI affordances (perceived creativity and perceived transparency) exert a buffering, moderating effect on the cognitive pathway. High levels of perceived creativity and transparency can weaken the inhibitory effect of positive reviews on cognitive involvement; however, this moderating effect is concentrated in the cognitive pathway and does not fully dispel consumers’ doubts regarding the authenticity of AI-generated positive reviews. Empathy cannot directly reduce psychological distance to the hotel. It must be fully mediated by a sequence involving psychological distance to the GAI and to the hotel to translate into booking intention.

This study constructs a model of the adoption of GAI review information, providing a more refined theoretical explanation of how users process AI-generated reviews. The findings offer clear directions for optimization for hotel booking platforms, hotel operators, and algorithm developers. Platforms should clearly label the time of AI-generated reviews and design user interfaces that guide users toward trusting information sources and foster a sense of closeness to hotel products; hotel operators should encourage guests to provide reviews that include specific emotional experiences, thereby offering rich emotional material for AI summarization; and algorithm design should, while maintaining information accuracy, moderately retain emotional vocabulary and experiential details, using affordance to support—rather than replace—the conveyance of emotions.

Although this study has made preliminary progress in elucidating the mechanisms underlying GAI reviews, limitations remain, including sample representativeness, the absence of a human-review control group, and limited explanatory power regarding psychological distance to the hotel. Future research can further enrich and validate the theoretical model of this study through cross-group and cross-cultural replication, the design of human-versus-AI review-comparison experiments, and the incorporation of multidimensional psychological distance cues, such as spatial and social distance.

## Figures and Tables

**Table 1:** Descriptive statistics of survey respondents (*N* = 649).

Characteristics	Levels	Frequency	Percentage (%)
Gender	Male	196	30.2
	Female	453	69.8
Age	18–29	282	43.5
	30–39	324	49.9
	40–49	28	4.3
	50 or above	15	2.3
Education	High school Degree or below	11	1.7
	Bachelor’s Degree	490	75.5
	Master’s Degree	142	21.9
	Doctor’s Degree	6	0.9

**Table 2:** Construct validity and reliability.

Variable	Items	Factor Loading	CA	CR	AVE
Perceived Creativity (PC)	PC1	0.925	0.879	0.925	0.805
	PC2	0.913			
	PC3	0.853			
Perceived Transparency (PT)	PT1	0.910	0.848	0.909	0.769
	PT2	0.916			
	PT3	0.800			
Cognitive Involvement (CI)	CI1	0.756	0.737	0.834	0.559
	CI2	0.806			
	CI3	0.788			
	CI4	0.627			
Affective Involvement (AI)	AI1	0.806	0.841	0.887	0.613
	AI2	0.762			
	AI3	0.796			
	AI4	0.847			
	AI5	0.695			
Cognitive Empathy (CE)	CE1	0.700	0.767	0.851	0.589
	CE2	0.794			
	CE3	0.778			
	CE4	0.795			
Affective Empathy (AE)	AE1	0.822	0.805	0.872	0.632
	AE2	0.830			
	AE3	0.792			
	AE4	0.731			
Psychological Distance to the GAI (AIPD)	AIPD1	0.958	0.843	0.906	0.763
	AIPD2	0.790			
	AIPD3	0.865			
Psychological Distance to the hotel (HPD)	HPD1	0.651	0.894	0.923	0.707
	HPD2	0.892			
	HPD3	0.863			
	HPD4	0.866			
	HPD5	0.905			
Booking Intention (BI)	BI1	0.977	0.977	0.983	0.936
	BI2	0.961			
	BI3	0.959			
	BI4	0.972			

*Note:* CA, Cronbach’s Alpha; CR, composite reliability; AVE, average variance extracted.

**Table 3:** Heterotrait–Monotrait ratio (HTMT).

Construct	AE	AI	AIPD	CE	CI	BI	PC	HPD	PT
AE	—								
AI	0.683	—							
AIPD	0.645	0.706	—						
CE	0.658	0.534	0.506	—					
CI	0.590	0.533	0.548	0.424	—				
BI	0.120	0.173	0.062	0.058	0.017	—			
PC	0.406	0.659	0.436	0.328	0.341	0.089	—		
HPD	0.091	0.297	0.188	0.113	0.121	0.695	0.182	—	
PT	0.358	0.475	0.370	0.289	0.251	0.080	0.520	0.146	—

*Note:* AE, affective empathy; AI, affective involvement; AIPD, psychological distance to the GAI; CE, cognitive empathy; CI, cognitive involvement; BI, booking intention; PC, perceived creativity; HPD, psychological distance to the hotel; PT, perceived transparency.

**Table 4:** Hypothesis test results.

Hypotheses	Path			Standardized Coefficient	T-Value	Results
H1	TD	→	CI	0.165	2.218 *	Supported
H2	VA	→	CI	−0.161	2.139 *	Supported
H3	VA	→	AI	0.194	3.068 **	Supported
H4	CI	→	CE	0.327	6.943 ***	Supported
H5	AI	→	AE	0.561	16.049 ***	Supported
H6	CE	→	HPD	0.053	1.117	Not Supported
H7	CE	→	AIPD	0.182	3.852 ***	Supported
H8	AE	→	HPD	−0.054	1.190	Not Supported
H9	AE	→	AIPD	0.444	9.707 ***	Supported
H10	AIPD	→	HPD	0.171	3.950 ***	Supported
H11	AIPD	→	BI	−0.055	1.864	Not Supported
H12	HPD	→	BI	0.674	33.678 ***	Supported
H13a	PC × VA	→	CI(+)	0.182	2.071 *	Supported
H13b	PC × VA	→	AI(+)	0.095	1.128	Not Supported
H14a	PT × VA	→	CI(+)	0.230	2.652 **	Supported
H14b	PT × VA	→	AI(+)	−0.111	1.304	Not Supported

*Note:* TD, temporal disclosure; PC, perceived creativity; CI, cognitive involvement; AI, affective involvement; CE, cognitive empathy; AE, affective empathy; AIPD, psychological distance to the GAI; HPD, psychological distance to the hotel; BI, booking intention; PT, perceived transparency; VA, review valence. * *p* < 0.05; ** *p* < 0.01; *** *p* < 0.001.

**Table 5:** Results of bootstrap analysis for mediation effects.

Paths	β	95% CI (LLCI, ULCI)
TD → CI → CE	0.054 *	[0.005, 0.109]
(Cognitive)		
VA → CI → CE	−0.053 *	[−0.103, −0.006]
(Cognitive)		
VA → AI → AE	0.109 **	[0.039, 0.177]
(Affective)		
CI → CE → AIPD	0.059 **	[0.027, 0.104]
(Cognitive)		
AI → AE → AIPD	0.249 ***	[0.185, 0.317]
(Affective)		
CE → AIPD → HPD	0.031 **	[0.013, 0.055]
AE → AIPD → HPD	0.076 ***	[0.037, 0.119]
AIPD → HPD → BI	0.115 ***	[0.058, 0.173]
VA → AI → AE → AIPD → HPD → BI	0.006 *	[0.001, 0.010]
(Affective)		
VA → CI → CE → AIPD → HPD → BI	−0.001	[−0.002, −0.000]
(Cognitive)		
TD → CI → CE → AIPD → HPD → BI	0.001	[0.000, 0.003]
(Cognitive)		

*Note:* * *p* < 0.05; ** *p* < 0.01; *** *p* < 0.001. *Abbreviations:* TD, temporal disclosure; VA, review valence; CI, cognitive involvement; AI, affective involvement; CE, cognitive empathy; AE, affective empathy; AIPD, psychological distance to the GAI; HPD, psychological distance to the hotel; BI, booking intention.

**Figure A1:**
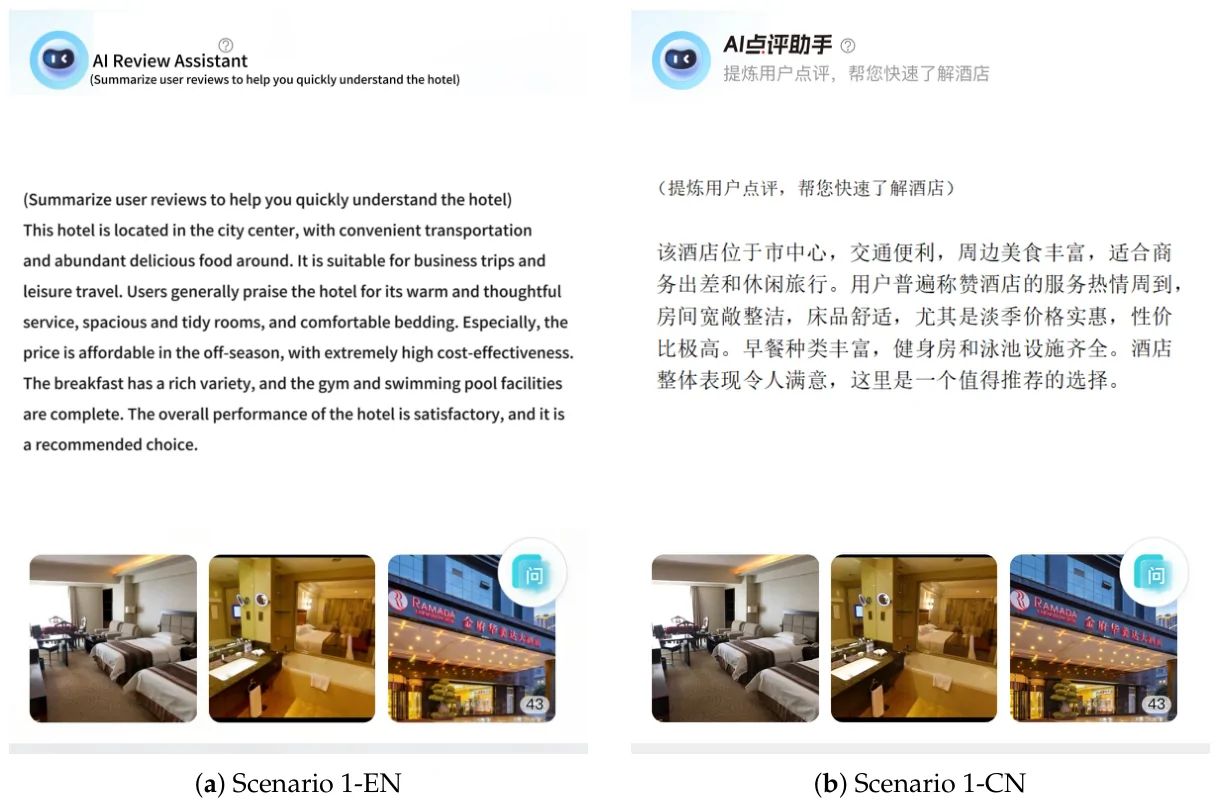
(**a**) Scenario 1 in English. (**b**) Scenario 1 in Chinese.

**Figure A2:**
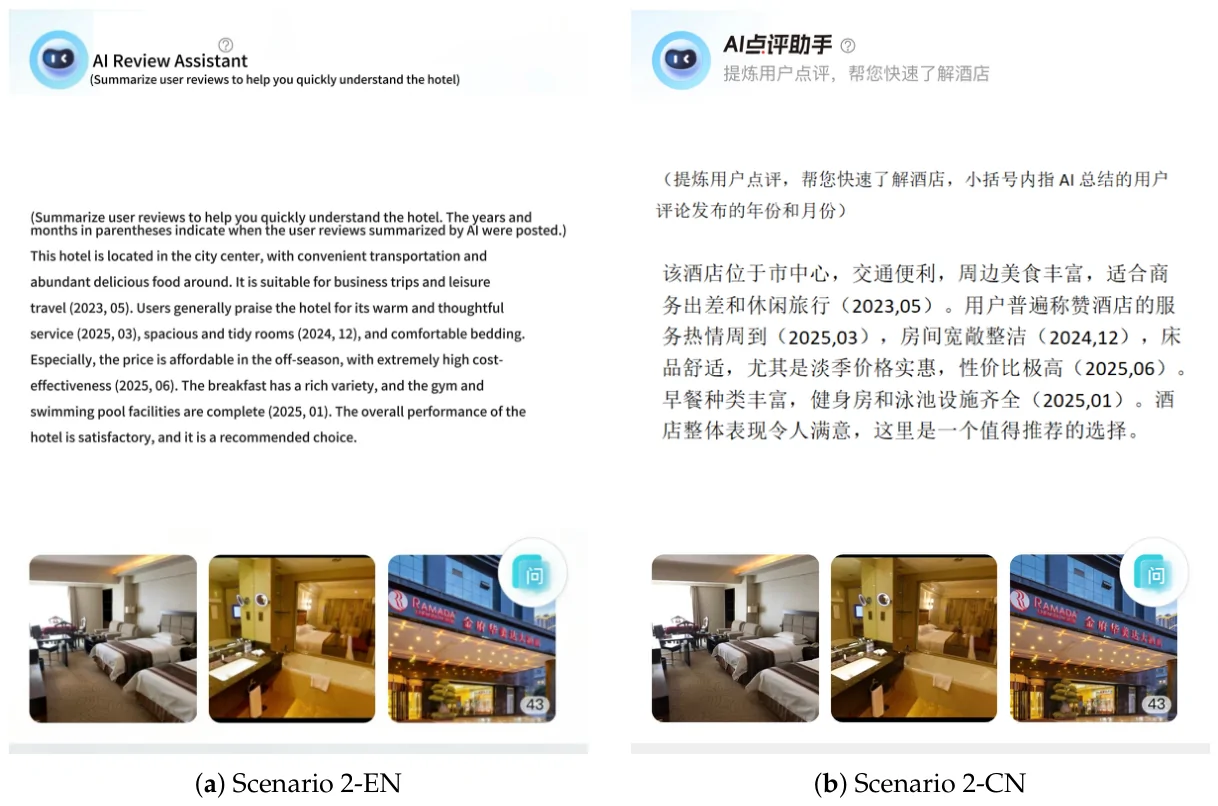
(**a**) Scenario 2 in English. (**b**) Scenario 2 in Chinese.

**Figure A3:**
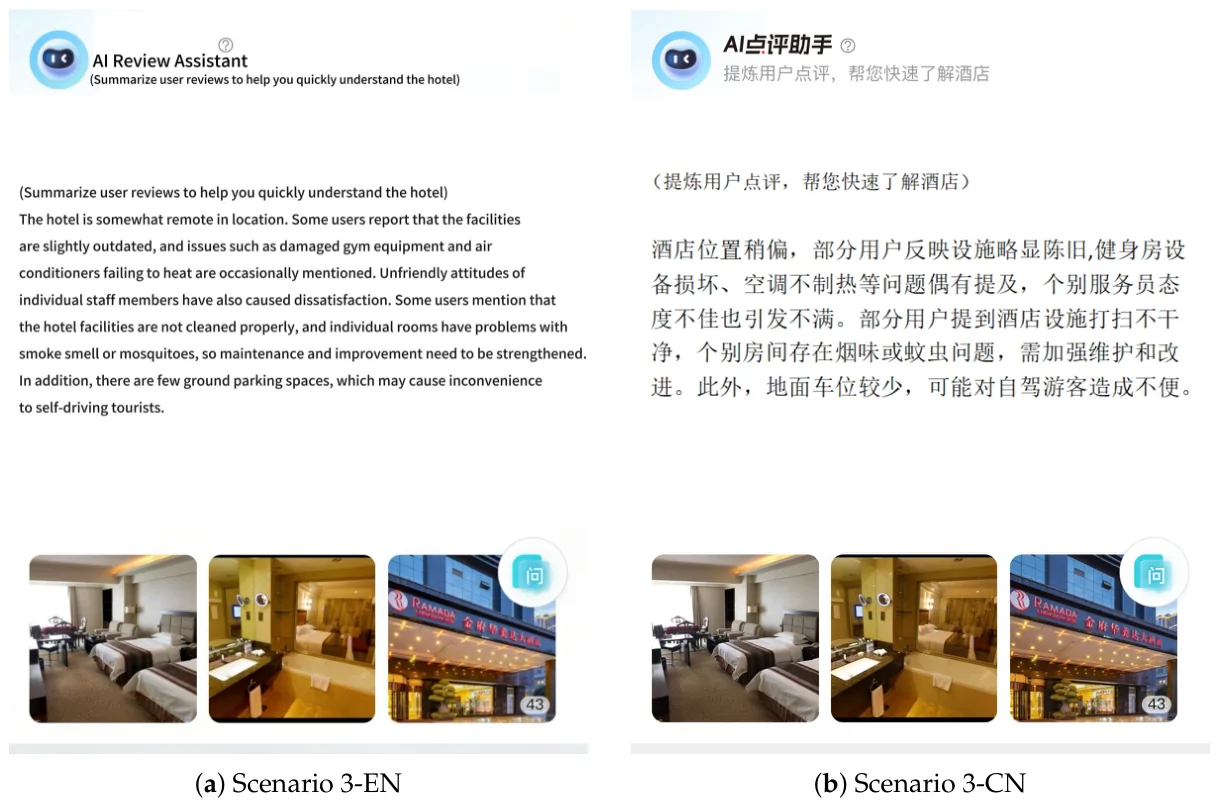
(**a**) Scenario 3 in English. (**b**) Scenario 3 in Chinese.

**Figure A4:**
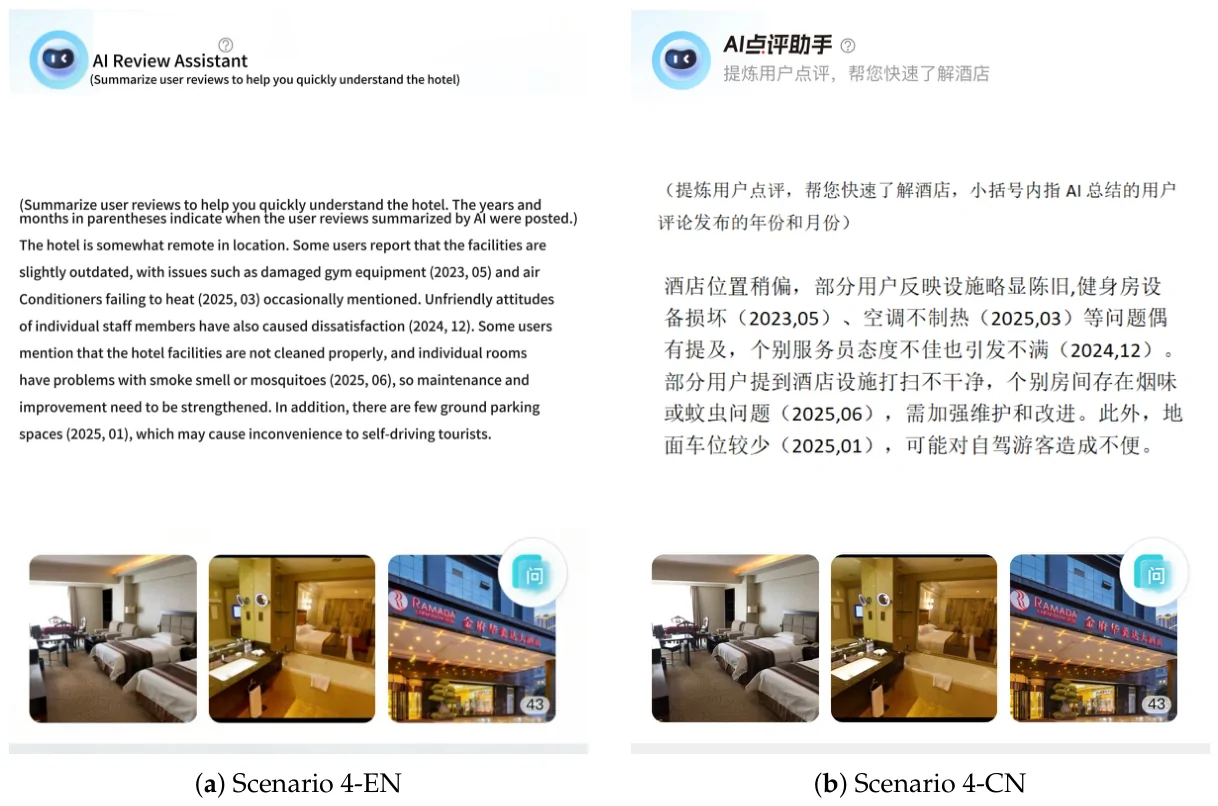
(**a**) Scenario 4 in English. (**b**) Scenario 4 in Chinese.

## Data Availability

The data that support the findings of this study are available from the corresponding authors upon reasonable request due to privacy.

## Supplementary Materials

The following supporting information can be downloaded at: https://www.mdpi.com/article/10.3390/bs16081417/s1, Supplementary File: Original Chinese questionnaire used in the formal survey, including all measurement items corresponding to Table A1 in the main text.

## Appendix A. Measurement Details

**Table A1:** Measurement details.

Construct and Measurement Items (1–7 Likert Scale)	Source of Measurement
**Booking Intention**	(D. J. Kim et al., 2008; R. Zhou & Tong, 2022)
BI1: I am likely to book this hotel.	
BI2: I am likely to recommend this hotel to my friends.	
BI3:I am likely to make another booking if I need this hotel.	
BI4: I am likely to consider booking this hotel after reading the AI review.	
**Perceived Transparency**	(Yu & Li, 2022)
PT1: I can access a great deal of information which explaining how the AI system works.	
PT2: I can see plenty of information about the AI system’s inner logic.	
PT3: I feel that the amount of the available information regarding the AI system’s reasoning is large.	
**Psychological Distance to the GAI**	(H. Chen et al., 2024; R. Chen & Yan, 2025)
AIPD1: I’m familiar with AI reviews.	
AIPD2: I think AI reviews are very similar to human reviews.	
AIPD3: Psychologically, I feel close to AI reviews.	
**Psychological Distance to the Hotel**	(H. Chen et al., 2024; R. Chen & Yan, 2025)
HPD1: I am familiar with the hotel that the AI reviewed.	
HPD2: Psychologically, I feel close to the hotel.	
HPD3: I think I can freely share my thoughts and feelings with the hotel.	
HPD4: I believe my ideas will be noticed by the hotel managers.	
HPD5: If I tell the hotel my problem, I think they can offer a solution or show concern.	
**Affective Involvement**	(J. M. Kang et al., 2015)
AI1: I think AI reviews are interesting.	
AI2: I think AI reviews are appealing.	
AI3: I think AI reviews are fascinating.	
AI4: I think AI reviews are exciting.	
AI5: I think AI reviews are involving.	
**Cognitive Involvement**	(J. M. Kang et al., 2015)
CI1: I think AI reviews are important.	
CI2: I think AI reviews are relevant.	
CI3: I think AI reviews are valuable.	
CI4: I think AI review means a lot to me.	
**Cognitive empathy**	(Vossen et al., 2015)
CE1: I understood the feelings expressed by AI reviews.	
CE2: I knew what the AI reviews felt emotionally.	
CE3: I could identify the feelings of AI reviews.	
CE4: I can easily judge the feel of AI reviews.	
**Affective empathy**	(Vossen et al., 2015)
AE1: The feelings of the AI reviews transferred to me.	
AE2: I felt the same way as AI reviews.	
AE3: I experienced the same emotions as the AI reviews.	
AE4: When AI review is angry about a product or service I get angry.	
**Perceived creativity**	(Casalo et al., 2021)
PC1: The AI review is revolutionary.	
PC2: The AI review is original.	
PC3: The AI review is unconventional.	

## Appendix B. Experimental Scenarios

Scenario 1—(non-disclosure × Positive) First, imagine that you are a traveler who is in urgent need of finding a suitable hotel to stay in. You click on the page of user reviews in the hotel platform. Below are the real AI-generated review from online hotel booking platforms. Please read carefully:

Scenario 2—(disclosure × Positive) First, imagine that you are a traveler who is in urgent need of finding a suitable hotel to stay in. You click on the page of user reviews in the hotel platform. Below are the real AI-generated review from online hotel booking platforms. Please read carefully:

Scenario 3—(non-disclosure × Negative) First, imagine that you are a traveler who is in urgent need of finding a suitable hotel to stay in. You click on the page of user reviews in the hotel platform. Below are the real AI-generated review from online hotel booking platforms. Please read carefully:

Scenario 4—(disclosure × Negative) First, imagine that you are a traveler who is in urgent need of finding a suitable hotel to stay in. You click on the page of user reviews in the hotel platform. Below are the real AI-generated review from online hotel booking platforms. Please read carefully:

## Appendix C. Analysis of Sample Heterogeneity

This study employed a permutation-based multigroup analysis (MGA) method to examine the intergroup robustness of the theoretical model across the two demographic dimensions of gender and age. The analysis followed the standard procedure for multigroup comparisons in Partial Least Squares Structural Equation Modeling (PLS-SEM): First, the intergroup equivalence of the measurement instruments was verified using the Multigroup Measurement Invariance Test (MICOM Step 2). Upon confirming measurement invariance, intergroup differences in path coefficients were further compared.

### Appendix C.1. Test for Heterogeneity by Gender Group

In this study, the sample was divided into male (N = 196) and female (N = 453) groups, and tests for composite invariance were conducted for all latent variables. The results showed that the permutation *p*-values for all constructs were greater than the critical threshold of 0.05, ranging from 0.117 to 0.982. This indicates that the measurement model in this study exhibits full composite invariance between the male and female groups; the conceptual content and factor loadings of the measurement instrument remain consistent across different gender groups; and subsequent between-group comparisons of path coefficients are statistically valid, with no measurement scale bias. The results of the composite invariance tests are presented in Table A2.

**Table A2:** Measurement Invariance of Composite Models and Step 2 Results (Male vs. Female).

Construct	Original	5.0%	Permutation
		Quantile	*p*-Value
AE	0.999	0.997	0.642
AI	1	0.999	0.949
AIPD	0.999	0.998	0.202
CE	0.998	0.991	0.618
CI	1	0.985	0.982
BI	1	1	0.57
PC	1	0.999	0.402
HPD	1	0.999	0.664
PT	1	0.995	0.648
TD	1	1	0.117
VA	1	1	0.156

*Note:* All permutation *p*-values > 0.05, indicating compositional invariance established. PC, perceived creativity; CI, cognitive involvement; AI, affective involvement; CE, cognitive empathy; AE, affective empathy; AIPD, psychological distance to the GAI; HPD, psychological distance to the hotel; BI, booking intention; PT, perceived transparency.

This study conducted multi-group comparisons of path coefficients. The results showed that most of the main-effect paths in the theoretical model did not differ significantly between the male and female groups (p>0.05), indicating that the model’s core logic is robust across genders. Only the path between H8 and H14b showed a statistically significant difference between the two groups. Specifically, in the path from affective empathy to psychological distance to the hotel (H8), the path coefficient for the male group was β=0.097, while that for the female group was β=−0.102; the between-group difference (p=0.048) reached statistical significance. This result indicates that the direction of the effect of affective empathy on psychological distance to the hotel differs between the two genders; however, the independent path coefficients for both groups did not reach the significance level, consistent with the finding that H8 was not significant in the full sample. Furthermore, regarding the moderating effect of perceived transparency on the influence of review valence on affective involvement (H14b), the moderating effect for the male group was β=−0.481, while for the female group, it was β=0.005; the difference between groups was p=0.011, reaching the level of significance. The results indicate that the moderating effect of perceived transparency on the relationship between review valence and affective involvement differs by gender. Among men, high transparency weakens the positive influence of review valence on affective involvement, whereas this moderating effect is virtually nonexistent among women.

The between-group differences for the remaining paths did not reach statistical significance, indicating that the core conclusions of this study are not influenced by gender and are universally applicable to both male and female travelers. The results of the heterogeneity test for gender groups are presented in Table A3.

**Table A3:** Multigroup Analysis Results (Male vs. Female).

Hypotheses	Path		Male (β)	Female (β)	Difference	Permutation *p*-Value
H1	TD	→ CI	−0.003	0.224	−0.227	0.161
H2	VA	→ CI	−0.042	−0.213	0.170	0.295
H3	VA	→ AI	0.214	0.187	0.027	0.846
H4	CI	→ CE	0.425	0.292	0.133	0.213
H5	AI	→ AE	0.587	0.561	0.026	0.735
H6	CE	→ HPD	−0.051	0.071	−0.122	0.251
H7	CE	→ AIPD	0.069	0.221	−0.152	0.158
H8	AE	→ HPD	0.097	−0.102	0.199	0.048 *
H9	AE	→ AIPD	0.414	0.472	−0.057	0.563
H10	AIPD	→ HPD	0.163	0.188	−0.025	0.795
H11	AIPD	→ BI	0.004	−0.079	0.083	0.195
H12	HPD	→ BI	0.689	0.667	0.022	0.608
H13a	PC × VA	→ CI	0.170	0.193	−0.023	0.903
H13b	PC × VA	→ AI	0.327	0.016	0.311	0.098
H14a	PT × VA	→ CI	0.042	0.284	−0.242	0.211
H14b	PT × VA	→ AI	−0.481	0.005	−0.485	0.011 *

*Note*: PC, perceived creativity; CI, cognitive involvement; AI, affective involvement; CE, cognitive empathy; AE, affective empathy; AIPD, psychological distance to the GAI; HPD, psychological distance to the hotel; BI, booking intention; PT, perceived transparency; VA, review valence; TD, temporal disclosure. * p<0.05.

### Appendix C.2. Test for Heterogeneity by Age Group

Additionally, regarding the age dimension, this study divided the sample into a Younger Group (ages 18–29, N = 282) and a Prime-Age Group (ages 30 and older, N = 367) to conduct combination invariance tests. The results show that the *p*-values for the substitution tests of all constructs were greater than 0.05, ranging from 0.084 to 0.661. These findings indicate that the measurement model exhibits compositional invariance across the two age groups; age differences do not lead to systematic bias in construct measurement, and the results of the multi-group comparisons are valid. Specific results are presented in Table A4.

This study conducted multi-group comparisons of path coefficients. The between-group differences in the age dimension were more pronounced than those in the gender dimension; significant between-group differences were found in four paths, reflecting divergent information processing patterns regarding GAI hotel reviews among different age groups. Specifically, the between-group difference in the path from review valence to cognitive involvement (H2) was significant (p=0.017). The path coefficient for the Younger group was β=−0.354, while that for the Prime-Age group was β=0.001. This indicates that cognitive involvement among individuals aged 30 and older is minimally influenced by the positive or negative valence of reviews, and their information processing tends to be more rational and stable. In the path from psychological distance to the GAI to psychological distance to the hotel (H10), the between-group difference was significant (p=0.007). The path coefficient for the Younger group was β=0.294, while that for the Prime-Age group was β=0.064. This suggests that younger individuals are more likely to transfer their trust in and affinity for GAI technology to the hotel described in the reviews, resulting in a stronger trust transfer effect; in contrast, older individuals distinguish more clearly between the information source and the decision object, leading to a weaker cross-object transfer of psychological connections. The between-group difference in the effect of psychological distance to GAI on booking intention (H11) was *p* < 0.001, indicating extreme significance. The path coefficient for the Younger group was β=−0.176, and for the Prime-Age group, it was β=0.055. Combined with the finding that H11 was not significant in the full sample, this confirms that the pattern—where psychological distance to GAI does not directly drive booking intention—holds consistently across the entire sample; after grouping, the younger group even exhibited a slight negative trend, suggesting that younger users may harbor a latent wariness toward AI.

**Table A4:** Measurement Invariance of Composite Models and Step 2 Results (Younger Group (aged 18–29) vs. Prime-Age Group (aged 30 and above)).

Construct	Original	5.0%	Permutation
		Quantile	*p*-Value
AE	0.999	0.997	0.427
AI	0.999	0.999	0.35
AIPD	1	0.998	0.546
CE	0.998	0.992	0.661
CI	0.997	0.988	0.477
BI	1	1	0.186
PC	1	0.999	0.361
HPD	1	0.999	0.556
PT	0.999	0.995	0.513
TD	1	1	0.084
VA	1	1	0.089

*Note:* All permutation *p*-values > 0.05, indicating compositional invariance established. PC, perceived creativity; CI, cognitive involvement; AI, affective involvement; CE, cognitive empathy; AE, affective empathy; AIPD, psychological distance to the GAI; HPD, psychological distance to the hotel; BI, booking intention; PT, perceived transparency.

Regarding the moderating effect of perceived creativity on the influence of review valence on cognitive involvement (H13a), the between-group difference was p=0.044, which was significant. The moderating effect for the Younger group was β=0.317, while for the Prime-Age group, it was β=−0.035. This indicates that the buffering moderating effect of GAI-perceived creativity is significant only among the younger group. In contrast, cognitive processing among the older group is less influenced by perceived creativity. Notably, in the core path of the model, the path where psychological distance to the hotel influences booking intention (H12) exhibited a significant positive effect in both groups, and the between-group difference was not significant (p=0.127). This indicates that bridging the psychological distance between consumers and the hotel can significantly enhance booking intention—a finding that stands as the most consistent core conclusion of this study, unaffected by differences across age groups. The results of the heterogeneity test for age groups are presented in Table A5.

**Table A5:** Multigroup Analysis Results (Younger Group (aged 18–29) vs. Prime-Age Group (aged 30–39 and above)).

Hypotheses	Path		Younger (β)	Prime-Age (β)	Difference	Permutation *p*-Value
H1	TD	→ CI	0.076	0.253	−0.178	0.244
H2	VA	→ CI	−0.354	0.001	−0.355	0.017 *
H3	VA	→ AI	0.097	0.307	−0.210	0.095
H4	CI	→ CE	0.307	0.351	−0.044	0.669
H5	AI	→ AE	0.547	0.561	−0.015	0.842
H6	CE	→ HPD	−0.016	0.135	−0.151	0.118
H7	CE	→ AIPD	0.232	0.138	0.094	0.348
H8	AE	→ HPD	−0.008	−0.122	0.114	0.231
H9	AE	→ AIPD	0.452	0.420	0.033	0.723
H10	AIPD	→ HPD	0.294	0.064	0.229	0.007 **
H11	AIPD	→ BI	−0.176	0.055	−0.231	<0.001 ***
H12	HPD	→ BI	0.722	0.661	0.062	0.127
H13a	PC × VA	→ CI	0.317	−0.035	0.352	0.044 *
H13b	PC × VA	→ AI	0.134	−0.029	0.163	0.341
H14a	PT × VA	→ CI	0.161	0.306	−0.145	0.414
H14b	PT × VA	→ AI	−0.013	−0.189	0.176	0.304

*Note*: PC, perceived creativity; CI, cognitive involvement; AI, affective involvement; CE, cognitive empathy; AE, affective empathy; AIPD, psychological distance to the GAI; HPD, psychological distance to the hotel; BI, booking intention; PT, perceived transparency; VA, review valence; TD, temporal disclosure. * p<0.05; ** p<0.01; *** p<0.001.

### Appendix C.3. Overall Conclusions from the Analysis of Sample Heterogeneity

First, the measurement tools demonstrate cross-group stability. Both gender and age subgroups passed combined measurement invariance tests, indicating that the scales and constructs used in this study have consistent meanings across population groups and that the measurement basis for the study’s conclusions is reliable. Second, gender has a negligible effect on the core model. The logic of the main effects in the theoretical model is highly consistent across male and female groups, with differences appearing only in a few marginal moderating paths; thus, the overall conclusions are universally applicable across genders. Third, age is an important boundary condition. Younger participants are more sensitive to informational cues in GAI reviews; their cognitive and affective responses are more easily influenced by review valence and GAI affordance, and they exhibit a stronger trust transfer effect. Older participants, in contrast, have a more stable information-processing style and rely less on technical cues. These findings also provide a basis for segmented, differentiated platform operations.

In summary, although the sample exhibits some concentration in terms of gender and age distribution, the core theoretical model remains robust across major demographic groups, indicating that the sample structure did not systematically distort the main conclusions of this study.
