# Supplementary material for: How GAI Shapes Travelers’ Booking Decisions: The Influence of Source Disclosure and Valence of Creative Reviews
Source: Behav Sci (Basel). 2026 Aug 18;16(8):1417. doi: 10.3390/bs16081417 (PMC13509170; doi:10.3390/bs16081417)
Supplement: Supplementary file 1 [file behavsci-16-01417-s001.zip › behavsci-4365911-supplementary.pdf]

Q1 请判断以上AI评论对该酒店是什么态度倾向 [单选]

○ 推荐

○ 不推荐

Q2 以上AI评论是否标注了评论来源时间 [单选]

○ 是

○ 否

VA （非常不同意=1，非常同意= 7）：

Q1 AI评论**推荐**此产品。 [单选]

○ 1

○ 2

○ 3

○ 4

○ 5

○ 6

○ 7

Q2 AI评论对这个产品**很满意**。 [单选]

○ 1

○ 2

○ 3

○ 4

○ 5

○ 6

○ 7

Q3 AI评论**不推荐**这个产品。 [单选]

○ 1

○ 2

○ 3

○ 4

○ 5

○ 6

○ 7

Q4 AI评论对这个产品**不满意**。 [单选]

○ 1

○ 2

○ 3

○ 4

○ 5

○ 6

○ 7

BI （非常不同意=1，非常同意= 7）：

Q1 我可能会预订这家酒店。 * [单选]

○ 1
○ 2
○ 3
○ 4
○ 5
○ 6
○ 7

Q2 我可能会向我的朋友推荐这家酒店。 * [单选]

○ 1
○ 2
○ 3
○ 4
○ 5
○ 6
○ 7

Q3 如果我需要入住酒店，我未来可能会再预订这家。 * [单选]

○ 1
○ 2
○ 3
○ 4
○ 5
○ 6
○ 7

Q4 在阅读了AI评论后，我可能会考虑预订该酒店。 * [单选]

○ 1
○ 2
○ 3
○ 4
○ 5
○ 6
○ 7

PT （非常不同意=1，非常同意= 7）：

Q1 我可以获得大量解释人工智能系统如何工作的信息。 * [单选]

○ 1
○ 2
○ 3
○ 4

○ 5
○ 6
○ 7

Q2 我可以看到很多关于人工智能系统内部逻辑的信息。 * [单选]
○ 1
○ 2
○ 3
○ 4
○ 5
○ 6
○ 7

Q3 我感觉人工智能系统推理的可用信息量很大。 * [单选]
○ 1
○ 2
○ 3
○ 4
○ 5
○ 6
○ 7

AIPD （非常不同意=1，非常同意= 7）:

Q1 我对AI评论了解了 * [单选]
○ 1
○ 2
○ 3
○ 4
○ 5
○ 6
○ 7

Q2 我认为AI评论和人类评论很相似 * [单选]
○ 1
○ 2
○ 3
○ 4
○ 5
○ 6
○ 7

Q3 从心理上来说，我觉得AI评论很亲近 * [单选]
○ 1

○ 2
○ 3
○ 4
○ 5
○ 6
○ 7

HPD （非常不同意=1，非常同意= 7）:

Q1 我对AI评论的这家酒店熟悉了 * [单选]

○ 1
○ 2
○ 3
○ 4
○ 5
○ 6
○ 7

Q2 从心理上，我觉得自己和这家酒店很亲近 * [单选]

○ 1
○ 2
○ 3
○ 4
○ 5
○ 6
○ 7

Q3 我认为我可以自由地与酒店分享我的想法和感受 * [单选]

○ 1
○ 2
○ 3
○ 4
○ 5
○ 6
○ 7

Q4 我相信我的想法会被酒店注意到 * [单选]

○ 1
○ 2
○ 3
○ 4
○ 5
○ 6
○ 7

Q5 如果我告诉酒店我的问题，我认为他们可以提供解决方案或表示关心 * [单选]

○ 1
○ 2
○ 3
○ 4
○ 5
○ 6
○ 7

AI （非常不同意=1，非常同意= 7）:

Q1 我认为AI评论是有趣的 * [单选]

○ 1
○ 2
○ 3
○ 4
○ 5
○ 6
○ 7

Q2 我认为AI评论是有感染力的 * [单选]

○ 1
○ 2
○ 3
○ 4
○ 5
○ 6
○ 7

Q3 我认为AI评论是有吸引力的 * [单选]

○ 1
○ 2
○ 3
○ 4
○ 5
○ 6
○ 7

Q4 我认为AI评论是令人兴奋的 * [单选]

○ 1
○ 2
○ 3
○ 4

○ 5
○ 6
○ 7

Q5 我认为AI评论是令人有参与感的 * [单选]

○ 1
○ 2
○ 3
○ 4
○ 5
○ 6
○ 7

CI （非常不同意=1，非常同意= 7）：

Q1 我认为AI评论是重要的 * [单选]

○ 1
○ 2
○ 3
○ 4
○ 5
○ 6
○ 7

Q2 我认为AI评论是与我相关的 * [单选]

○ 1
○ 2
○ 3
○ 4
○ 5
○ 6
○ 7

Q3 我认为AI评论是有价值的 * [单选]

○ 1
○ 2
○ 3
○ 4
○ 5
○ 6
○ 7

Q4 我认为AI评论是有意义的 * [单选]

○ 1

○ 2
○ 3
○ 4
○ 5
○ 6
○ 7

CE （非常不同意=1，非常同意= 7）：
Q1 我能理解AI评论表达的感受 * [单选]
○ 1
○ 2
○ 3
○ 4
○ 5
○ 6
○ 7

Q2 我知道AI评论的情绪感受 * [单选]
○ 1
○ 2
○ 3
○ 4
○ 5
○ 6
○ 7

Q3 我能够识别出AI评论的感受 * [单选]
○ 1
○ 2
○ 3
○ 4
○ 5
○ 6
○ 7

Q4 我能轻易地判断AI评论的感受 * [单选]
○ 1
○ 2
○ 3
○ 4
○ 5
○ 6
○ 7

AE （非常不同意=1，非常同意= 7）：

Q1 AI评论的感受转移到了我的身上 * [单选]

○ 1
○ 2
○ 3
○ 4
○ 5
○ 6
○ 7

Q2 我与AI评论有同样的感受 * [单选]

○ 1
○ 2
○ 3
○ 4
○ 5
○ 6
○ 7

Q3 我体验到了与AI评论相同的感情 * [单选]

○ 1
○ 2
○ 3
○ 4
○ 5
○ 6
○ 7

Q4 AI评论对产品或服务生气的时候，我也会感到生气 * [单选]

○ 1
○ 2
○ 3
○ 4
○ 5
○ 6
○ 7

PC （非常不同意=1，非常同意= 7）：

Q1 AI生成的评论是创新性的。 * [单选]

○ 1
○ 2
○ 3

○ 4
○ 5
○ 6
○ 7

Q2 AI生成的评论是独创的。 * [单选]

○ 1
○ 2
○ 3
○ 4
○ 5
○ 6
○ 7

Q3 AI生成的评论是非传统的。 * [单选]

○ 1
○ 2
○ 3
○ 4
○ 5
○ 6
○ 7
